# Impact of Motor-Cognitive Interventions on Selected Gait and Balance Outcomes in Older Adults: A Systematic Review and Meta-Analysis of Randomized Controlled Trials

**DOI:** 10.3389/fpsyg.2022.837710

**Published:** 2022-06-16

**Authors:** Kaja Teraz, Luka Šlosar, Armin H. Paravlić, Eling D. de Bruin, Uros Marusic

**Affiliations:** ^1^Institute for Kinesiology Research, Science and Research Centre Koper, Koper, Slovenia; ^2^Faculty of Sport, University of Ljubljana, Ljubljana, Slovenia; ^3^Faculty of Sports Studies, Masaryk University, Brno, Czechia; ^4^Institute of Human Movement Sciences and Sport, Department of Health Sciences and Technology, ETH Zurich, Zurich, Switzerland; ^5^Division of Physiotherapy, Department of Neurobiology, Care Sciences and Society, Karolinska Institute, Stockholm, Sweden; ^6^Department of Health Sciences, Alma Mater Europaea – ECM, Maribor, Slovenia

**Keywords:** motor-cognitive intervention, dual-task, elderly, mobility, postural control

## Abstract

**Background:**

Efficient performance of most daily activities requires intact and simultaneous execution of motor and cognitive tasks. To mitigate age-related functional decline, various combinations of motor and cognitive training have shown promising results. The aim of this systematic review and meta-analysis of randomized controlled trials (RCTs) was to evaluate the efficacy of different types of motor-cognitive training interventions (e.g., sequential and simultaneous) on selected functional outcomes in healthy older adults.

**Methods:**

Six online academic databases were used to retrieve eligible RCTs up to April 2021, following PRISMA guidelines and PICO criteria. A random-effects model was used for all meta-analyses conducted on selected functional outcomes: single- and dual-task gait speed, the Timed Up and Go Test (TUG), and Berg Balance Scale (BBS) score. Effect size (ES) was calculated as Hedges' g and interpreted as: *trivial*: <0.20, *small*: 0.20–0.60, *moderate*: 0.61–1.20, *large:* 1.21–2.00, *very large*: 2.01–4.00 or *extremely large* >4.00.

**Results:**

From 2,546 retrieved records, 91 RCTs were included for meta-analysis (*n* = 3,745 participants; 64.7–86.9 years). The motor-cognitive interventions included differed according to the type of training (e.g., sequential, simultaneous with additional cognitive task or exergame training. The results showed that motor-cognitive interventions can improve gait speed under single-task conditions (*small* ES = 0.34, *P* = 0.003). The effect of the intervention was moderated by the type of control group (*Q* = 6.203, *P* = 0.013): passive (*moderate* ES = 0.941, *P* = 0.001) vs. active controls (*trivial* ES = 0.153, *P* = 0.180). No significant effect was found for dual-task walking outcomes (*P* = 0.063). Motor-cognitive intervention had a positive effect on TUG (*small* ES = 0.42, *P* < 0.001), where the effect of intervention was moderated by control group [passive (*moderate* ES = 0.73, *P* = 0.001) vs. active (*small* ES = 0.20, *P* = 0.020)], but not by the type of training (*P* = 0.064). Finally, BBS scores were positively affected by motor-cognitive interventions (*small* ES = 0.59, *P* < 0.001) with however no significant differences between type of control group (*P* = 0.529) or intervention modality (*P* = 0.585).

**Conclusions:**

This study provides evidence for the effectiveness of various types of motor-cognitive interventions on performance-based measures of functional mobility in healthy older adults. With respect to significant effects, gait speed under single-task condition was improved by motor-cognitive interventions, but the evidence shows that this type of intervention is not necessarily more beneficial than motor training alone. On the other hand, motor-cognitive interventions are better at improving multicomponent tasks of dynamic balance and mobility function, as measured by the TUG. Because of substantial heterogeneity and the current limited availability of different types of interventions, the conclusions should be interpreted with caution.

## Introduction

Aging leads to a decline in physical and cognitive abilities, which has been associated with an increased incidence of falls (Lord et al., 1999; Masud and Robert, 2001; Ambrose et al., 2013). Falls occur when everyday tasks become too difficult (either physically or cognitively) and can lead to various injuries that later affect functioning in old age (Masud and Robert, 2001; Tinetti, 2003; Ambrose et al., 2013). Older adults typically struggle with tasks that must be performed simultaneously, such as using a cell phone and walking down the stairs or simultaneously observing the traffic and stepping off the sidewalk at the same time (Beurskens and Bock, 2012; MacPherson, 2018). This “ability to perform two tasks simultaneously” (MacPherson, 2018) is defined as dual-tasking. Dual-tasking is often challenging for older adults, but the underlying mechanism is not yet clear. Older people engage more cognitive control in mobility tasks (Marusic and Grosprêtre, 2018). This is partly due to age-related sensory impairments and partly due to lower automated motor and cognitive performance (Baltes and Lindenberger, 1997; Li and Lindenberger, 2002; Heuninckx et al., 2005; Wollesen and Voelcker-Rehage, 2014). Human attention is limited (Jiang and Kanwisher, 2003) and both physical and cognitive changes that occur in the brain during aging impair executive functions (Peters, 2006).

Appropriate training, whether motor or cognitive training, can slow down the decline of motor and cognitive functions (Allen et al., 2011; Schoene et al., 2013; Smith et al., 2015; Hortobágyi et al., 2016). Studies are describing different types of exercise for older people to improve mobility-related outcomes; motor training (Allen et al., 2011; Hortobágyi et al., 2016), cognitive training (e.g., Smith et al., 2015), and motor-cognitive dual-task training (e.g., Schoene et al., 2013). Recent systematic reviews have shown that motor (for a review see Plummer et al., 2015) and cognitive training (for a review see Marusic et al., 2018b) can have positive effects on mobility in older adults.

In 2010, two research groups conducted two separate pilot studies that indicated extensive transfer from cognitive training to mobility domain (Li et al., 2010; Verghese et al., 2010). After these two pilot trials, there were many other studies that confirmed this effect, which was also summarized in a meta-analysis (for review see Marusic et al., 2018b). In addition, the various types of non-physical interventions (e.g., cognitive training, motor imagery and action observation) can improve motor-related outcomes (Marusic et al., 2018a; Paravlic et al., 2018, 2019). The potential mechanisms of improved mobility performance after non-physical training sessions have been suggested by intertwined neural circuits and brain substrates involved in both cognitive (executive functions) and mobility processes (Marusic et al., 2018b).

The so-called motor-cognitive training is a type of dual-task training, i.e., it involves two different tasks (the motor task and the cognitive task) that can be performed simultaneously or sequentially (Herold et al., 2018), where one of the tasks specifically challenges motor functions and the other task challenges cognitive functions. In sequential training, the motor task (e.g., walking) and the cognitive task (e.g., solving tasks while sitting at a table and using a desktop computer) are separated (Herold et al., 2018). In simultaneous motor-cognitive training, both motor and cognitive exercises are executed at the same time (Lauenroth et al., 2016; Herold et al., 2018). This type of training can be divided into two types: (i) motor training with cognitive exercises that tend to be unrelated to motor task performance, and (ii) motor training in which successful physical task performance depends on cognitive ability (Herold et al., 2018). If the cognitive exercise appears to be more of a distractor, a simultaneous motor-cognitive training is performed with an additional cognitive task (e.g., cycling while counting backwards from 50 and subtracting 4 s). Conversely, simultaneous motor-cognitive training with a built-in cognitive task (e.g., exergame/exergaming or learning to dance) is conducted when the cognitive exercise fits the content of the intervention as a necessary task to successfully complete the training (Schott, 2015; Manser et al., 2021). Exergaming is defined as technology-based physical activities, such as playing video games, that require participants to be physically active or move in order to play the game. These games require the user to move their entire body to participate in virtual sports, group fitness exercises, or other interactive physical activities (American College of Sports Medicine., 2013).

The different types of motor-cognitive training (sequential, simultaneous with additional or incorporated cognitive task) have not been studied. Therefore, the combination of motor and cognitive intervention has recently gained scientific interest. Several reviews and intervention studies have already reported positive effects of motor-cognitive interventions on single- and dual-task walking and balance in both healthy and cognitively impaired older adults (Law et al., 2014; Fritz et al., 2015; Lauenroth et al., 2016; Zhu et al., 2016; Levin et al., 2017; Raichlen et al., 2020; Chen et al., 2021). However, a systematic investigation on the most effective of all motor-cognitive interventions (sequential, simultaneous with an additional or incorporated cognitive task) effecting gait and balance is not available. Our aim was to identify, summarize, and compare randomized controlled trials (RCTs) examining motor-cognitive intervention approaches vs. single or no training interventions in older adults on selected gait and balance functions.

## Methods

### Search Strategy

We performed a systematic literature search in six bibliographic databases, i.e., PubMed, Pedro, Cinahl, SportDiscus, and Scopus. In addition, we performed a literature search on Google Scholar. The search strategy included only terms related to or describing the intervention. Terms were combined with the Cochrane MEDLINE filter for controlled trials of interventions. Our search syntax was: (“motor-cognitive intervention” OR “dual-task” OR “motor-cognitive training” OR “physical-cognitive intervention” OR “motor-cognitive exercise” OR “exergames” OR “serious game” OR “active video game”) AND (“gait” OR “walk” OR “walking” OR “mobility” OR “balance” OR “posture”) AND (“elderly” OR “old” OR “older” OR “older adult” OR “older adults” OR “aging” OR “elder adults” OR “elders” OR “old-olds”). Search terms were adapted for use with other bibliographic databases in combination with database-specific filters for controlled trials, where these are available. Database searches were supplemented by the review of the authors files. We additionally reviewed the reference list of each included article. We included only studies published in English. When searching for articles, we did not set a time frame for publication. Nevertheless, all articles that met our inclusion criteria were published between 2009 and 2021. The searches were performed again just before the final analysis and additional studies were selected for inclusion. Titles and abstracts that did not meet the inclusion criteria were excluded from the list. The remaining full texts were screened by 3 reviewers (UM, KT, and LŠ). Ultimately, only randomized controlled trials that met the listed inclusion criteria were included.

### Selection Criteria

The strategy for the literature search followed the PRISMA (The Preferred Reporting Items for Systematic Reviews and Meta-Analyses) guidelines. This is an evidence-based minimum set of items for reporting in systematic reviews and meta-analyses. PRISMA is used as the basis for reporting systematic reviews with objectives. In addition, we included the Problem/Population, Intervention, Comparison, and Outcome (PICO) framework; it can help formulate the search strategy with set of key questions to efficiently find high- quality evidence:

*Population*: healthy and diseased^1^ older adults; the mean age of subjects was over 60 years.
*Intervention*: motor-cognitive approaches (sequential, simultaneous distractor, simultaneous incorporated).
*Comparison*: either passive control (neither cognitive nor motor training performed) or motor training groups.
*Outcome measures*: Gait speed under (m/s) a single- and a dual-task condition, balance performance as measured with Timed Up and Go test (sec) and Berg Balance Scale (points).

We included only randomized controlled trials (RCTs). The inclusion criteria were: (1) the type of outcome measure was gait speed under a single- and/or a dual-task, the Up and Go test and/or the Berg Balance Scale, (2) subjects who performed motor-cognitive interventions were compared with those who performed only motor interventions or subjects who were in the passive control group; (3) the mean age of subjects was greater than 60 years (4) studies in which the effect of interventions was of interest if data were available.

Initially, we included subjects classified as “healthy” older adults with no specific diseases diagnosed and individuals with different diseases such as Parkinson's disease, balance impairment, mild cognitive impairment, osteoporosis, dementia, diabetes mellitus, Alzheimer's disease, and studies, that included either patients after stroke or hospitalized patients or patients with a history of falls, or osteoarthritic patients with balance impairment or older adults who were classified as frail or adults with various motor and cognitive deficits or residents of long-term-care facilities or patients with severe neurocognitive disorders. Because we found a high degree of heterogeneity within the groups diagnosed with a particular deficit, we decided to exclude from further analysis all studies that included diseased individuals. However, we have left a summary of all studies included in the original analysis in Supplementary Tables 1–4.

### Screening Strategy

Three independent authors (KT, UM, and LŠ) conducted the search for available studies on a selected topic. The screening was performed in four steps. First, the titles were screened by the reviewers to determine whether they were suitable for our meta-analysis. Then, abstracts were assessed to determine whether the study topic met the selected inclusion and exclusion criteria. The inclusion criteria were selected as follows (as mentioned above) and are described in Table 1: the mean age of the participants was 60 years or more, the type of intervention was a motor-cognitive intervention, the comparison group was either passive or with included motor intervention, the outcomes of the studies were gait speed under a single- or a dual-task condition, TUG and BBS test and the study design was a RCT. The exclusion criteria were: no control group, irrelevant outcomes, unsuitable measurement of gait (e.g., measures were performed on a treadmill), inadequate results and unsuitable measurement of balance (e.g., measures were performed on a force plate). Third, the full text articles were read, the required information was selected and included (if appropriate) in the meta-analysis. Finally, the references of the included studies were reviewed for possible inclusion. If the full text of any paper was unavailable or the data of the study were incomprehensible (certain data on results were missing, e.g., standard deviation, or we were unable to deduce the value of the results from the reported data, e.g., data were reported in graphs), the corresponding author was contacted by email. Disagreements about the inclusion/exclusion of certain RCT were resolved by discussion or by a third person when no consensus could be reached (EdB, AP).

**Table 1:** Inclusion and exclusion criteria.

	**Inclusion criteria**
Study design	RCT
Language	English
Mean age of participants:	60 years or more
Type of intervention	Motor-cognitive intervention
Comparison group	Passive control group
	Active control group – motor training
Outcomes of the study	Gait speed under a single – task condition
	Gait speed under a dual- task condition
	Timed Up and Go test
	Berg Balance Scale test
	**Exclusion criteria**
	No control group
	Participants younger than 60 years
	Non-English language
	Unsuitable measurement of balance (e.g., force plate)
	Unsuitable measurement of gait (e.g., treadmill)
	Unsuitable motor-cognitive interventions (e.g., additionally added cognitive tasks during interventions, incorporated cognitive task that was not part of exergame intervention)

The Physiotherapy Evidence Database (PEDro) scale was used to assess the risk of bias and quality of included studies (Maher et al., 2003). This scale helps the reader to quickly assess whether a clinical trial presents reliable and meaningful results for use in clinical practice. Points are awarded only when a criterion is clearly met. In addition, points are awarded according to the specifics of the article and if the article meets those specifics (eligibility criteria were specified, subjects were randomly assigned to groups, assignment was concealed, the groups were similar at baseline regarding the most important prognostic indicators, there was blinding of all subjects, there was blinding of all therapist who administered the therapy, there was blinding of all assessors who measured at least one key outcome, measures of at least one key outcome were obtained from more than 85% of the subjects initially allocated to groups, all subjects from whom outcome measures were available received the treatment or control condition as allocated or, where this was not the case, data for at least one key outcome was analyzed by “intention to treat”, the results of between group statistical comparisons are reported for at least one key outcome). The quality assessment score was interpreted as follows: studies scoring 6–10 points on PEDro quality assessment were of “high quality”, studies scoring 4–5 of “fair quality” and studies scoring 0–3 of “poor quality”. The evaluation of the studies is available in Table 2.

**Table 2:** Quality assessment of included studies with healthy older adults according to PEDro scale.

**Study**	**Quality criteria**											**Quality score**
	**1**	**2**	**3**	**4**	**5**	**6**	**7**	**8**	**9**	**10**	**11**	
Bieryla and Dold (2013)	X	X	-	X	-	-	-	-	-	-	-	3
Bieryla (2016)	X	X	-	X	-	-	-	-	X	X	X	6
Bischoff et al. (2020)	X	X	-	X	-	-	X	-	-	X	X	5
de Bruin et al. (2013)	X	X	-	X	-	-	-	-	-	X	X	5
Chao et al. (2015)	X	X	-	X	-	-	-	X	-	X	X	6
Desjardins-Crepeau et al. (2016)	X	X	-	X	-	-	X	-	-	X	X	6
Eggenberger et al. (2015)	X	X	-	X	-	-	-	-	X	X	X	5
Eggenberger et al. (2016)	X	X	-	X	-	-	-	-		X	-	3
Falbo et al. (2016)	X	X	-	X	-	-	-	-	-	X	X	5
Franco et al. (2012)	X	X	-	X	-	-	-	X	-	X	X	6
Fraser et al. (2017)	X	X	-	X	-	-	-	-	-	X	X	5
Gallardo-Meza et al. (2022)	X	X	-	X	-	-	-	X	X	-	X	6
Gregory et al. (2016)	X	X	X	X	-	-	X	-	X	X	X	8
Gschwind et al. (2015)	-	X	-	X	-	-	-	X	X	X	X	6
Gschwind et al. (2015)	X	X	-	X	-	-	-	X	X	X	X	7
Hiyamizu et al. (2012)	X	X	X	X	-	-	X	-	X	X	X	8
Jardim et al. (2021)	X	-	-	X	-	-	-	X	X	X	X	6
Jehu et al. (2017)	X	X	-	X	-	-	-	X	-	X	X	6
Jorgensen et al. (2013)	X	X	-	X	-	-	X	X	X	X	X	8
Kao et al. (2018)	X	X	X	X	X	X	-	X	-	X	-	8
Karahan et al. (2015)	X	X	-	X	-	-	-	X	X	X	X	7
Kwok and Pua (2016)	X	X	X	X	-	-	X	-	-	X	X	7
Lai et al. (2013)	-	X	-	X	-	-	X	-	-	-	X	4
Lee et al. (2015)	X	X	-	X	-	-	X	X	X	X	X	8
Lee et al. (2018)	X	X	-	X	-	-	X	X	-	X	X	7
Maillot et al. (2012)	X	X	-	X	-	-	-	X	X	-	X	6
Medeiros et al. (2018)	X	X	-	X	-	-	-	X	X	X	X	7
Morat et al. (2019)	X	X	-	X	-	-	-	X	-	X	X	6
Nagano et al. (2016)	X	X	X	-	-	-	X	X	X	X	X	8
Nematollahi et al. (2016)	X	X	X	X	X	X	-	-	X	X	X	8
Ng et al. (2015)	X	X	X	X	-	-	X	X	X	X	X	9
Nishiguchi et al. (2015)	X	X	-	X	-	-	X	X	-	X	X	7
Norouzi et al. (2019)	X	X	X	X	-	-	-	X	X	X	X	8
Padala et al. (2012)	X	X	-	X	-	-	-	-	X	X	X	6
Park et al. (2015)	-	X	-	X	-	-	-	-	-	X	X	4
Phirom et al. (2020)	X	X	X	-	-	-	-	X	-	X	X	6
Pichierri et al. (2012)	X	X	-	X	-	-	-	-	-	X	X	5
Pluchino et al. (2012)	X	X	X	X	-	-	-	-	-	X	X	6
Plummer-D'Amato et al. (2012)	X	X	X	X	-	-	X	X	-	X	X	8
Pothier et al. (2018))	-	X	-	X	-	-	-	-	-	X	X	4
Raichlen et al. (2020)	X	X	-	X	-	-	X	-	X	X	X	7
Rendon et al. (2012)	X	X	-	X	-	-	X	-	X	X	X	7
Rezola-Pardo et al. (2019)	X	X	X	X	-	-	X	-	X	X	X	8
Sadeghi et al. (2021)	X	X	X	-	-	-	-	-	X	X	X	6
Salazar-González et al. (2015)	X	X	X	X	-	-	-	-	X	X	X	7
Sápi et al. (2019)	X	-	-	-	-	-	-	-	-	X	X	3
Sato et al. (2015)	-	X	-	X	-	-	-	X	-	X	X	6
Schättin et al. (2016)	X	X	-	X	-	-	-	X	-	X	X	6
Schoene et al. (2013)	X	X	X	X	-	-	X	X	-	X	X	8
Schwenk et al. (2014)	X	X	X	X	-	-	-	X	-	X	X	7
Sipilä et al. (2021)	X	X	-	X	-	-	X	X	X	X	X	8
Theill et al. (2013)	X	-	-	X	-	-	-	-	-	X	X	4
van het Reve and de Bruin (2014)	X	X	X	X	X	-	-	X	X	X	X	9
Yamada et al. (2011a)	X	X	X	X	-	-	X	X	-	X	X	8
Yamada et al. (2011b)	X	X	X	X	-	-	X	X	-	X	X	8
Yesilyaprak et al. (2016)	X	X	-	X	-	-	X	X	-	X	X	7
Yoo et al. (2013)	-	X	-	X	-	-	-	X	-	X	X	5
Wongcharoen et al. (2017)	X	X	X	X	-	-	X	X	-	X	-	7

^*^1-eligibility criteria were specified, 2-subjects were randomly allocated to groups, 3-allocation was concealed, 4-the groups were similar at baseline regarding the most important prognostic indicators, 5-there was blinding of all subjects, 6-there was blinding of all therapists who administered the therapy, 7-there was blinding of all assessors who measured at least one key outcome, 8-measures of at least one key outcome were obtained from more than 85% of the subjects initially allocated to groups, 9-all subjects from whom outcome measures were available received the treatment or control condition as allocated or, where this was not the case, data for at least one key outcome was analyzed by ≫intention to treat≪, 10-the results of between-group statistical comparisons are reported for at least one key outcome, the study provides both point measures and measures of variability for at least one key outcome.

All included studies were divided into three groups (Tables 3–5) according to the type of dual-task intervention they performed. According to Herold's definition (Herold et al., 2018), we divided the studies into those that can be performed simultaneously or sequentially. Furthermore, studies that included training with a dual-task performed simultaneously were further divided into studies that performed simultaneous motor-cognitive training with additional cognitive task or those who performed simultaneous motor-cognitive training with incorporated cognitive task. In the category of simultaneous motor-cognitive training with an incorporated cognitive task, there were very few studies that did not use exergaming. We excluded the studies that did not meet the definition of exergaming and formed a group with exergaming studies only. The third group is therefore called exergaming.

**Table 3:** Sequential motor-cognitive training.

**Study**	**Sample description**	**Experimental design and duration of trial period**	**Control design and duration of trial period**	**Outcomes and results**
Desjardins-Crepeau et al. (2016)	*N =* 76 (CON ST = 18, CON AR = 16, EXP ARdt = 22, EXP STdt = 20) Mean age_CONST_ = 72.5 ± 7.0 Mean age_CONAR_ = 70.9 ± 7.4 Mean age_EXPARdt_ = 72.7 ± 7.4 Mean age_EXPSTdt_ = 73.2 ± 6.3	ARdt = In addition to the aerobic and resistance training participants performed cognitive training on a PC (visual discrimination tasks performed separately and concurrently) STdt = In addition to stretching and toning training participants performed cognitive training on a PC (visual discrimination tasks performed separately and concurrently) PA training: 12 weeks: 2-times per week (60 min/trial) Cognitive training: 12 weeks: 1-time per week (60 min/trial)	AR = Aerobic exercises + passive computer lessons (excel, word) ST = Stretching exercises + passive computer lessons (excel, word) 12 weeks: 3-times per week (2x-physical exercise + 1x-passive computer lessons; 60 min/trial)	TUG (no improvement) Gait speed (6MWT) ↑ for CON and EXP group
Pothier et al. (2018)	*N =* 90 (CON ST = 18, CON AR = 21, EXP ARdt = 28, EXP STdt = 23) Mean age_CONST_ = 72.5 ± 6.9 Mean age_CONAR_ = 69.7 ± 6.5 Mean age_EXPARdt_ = 72.2 ± 7.0 Mean age_EXPSTdt_ = 74.2 ± 6.9	ARdt = In addition to the aerobic and resistance training participants performed cognitive training on a PC (number and shape discrimination tasks) STdt = In addition to stretching and toning training participants performed cognitive training on a PC (number and shape discrimination tasks) PA training: 12 weeks: 2-times per week (60 min/trial) Cognitive training: 12 weeks: 1-time per week (60 min/trial)	AR = Aerobic exercises + passive computer lessons (excel, word) ST = Stretching exercises + passive computer lessons (excel, word) 12 weeks: 3-times per week (2x-physical exercise + 1x-computer lessons; 60min/trial)	Gait speed - ↑ for both EXP group and CON AR
van het Reve and de Bruin (2014)	*N =* 145 (CON = 76, EXP = 69); Mean age_CON =_81.9 ± 6.3 Mean age_EXP_ = 81.1 ± 8.3	In addition to the strength and balance training participants performed CogniPlus program. PA training: 12 weeks: 2-times per week (40 min/trial) Cognitive training: 12 weeks: 3-times per week (10 min/trial)	Strength and balance training 12 weeks, 2-times per week (40 min/trial)	Gait speed (no improvement) ETGUG – ↑ for CON and EXP group
de Bruin et al. (2013)	*N =* 14 (CON = 7, EXP = 7); Mean age_CON =_75.0 ± 8.3 Mean age_EXP_ = 79.8 ± 6.9	In addition to the strength and balance training participants performed CogniPlus program PA training: 12 weeks, 2-times per week (45–60 min/trial) Cognitive training: 10 weeks: 3 to 5-times per week (10 min/trial)	Strength and balance training 12 weeks, 2-times per week (45–60 min/trial)	ETGUG - ↑ for CON and EXP
Ng et al. (2015)	*N =* 147 (CON passive = 50, CON motor = 48, EXP = 49) Mean age_CONpassive_ = 70.1 ± 5.0 Mean age_CONmotor_ = 70.3 ± 5.2 Mean age_EXP_ = 70.4 ± 4.7	In addition to the strength and balance training participants performed cognitive training (stimulate short term memory, enhance attention and information-processing skills and reasoning and problem solving abilities) PA training: 12 weeks: 2-times per week (90 min/trial) + 12 weeks home based exercises	CON passive = Passive group (only pre- and post-intervention assessment) CON motor = The exercise program was designed to improve strength and balance	Gait speed (6MWT) ↑ for CON motor
Fraser et al. (2017)	*N =* 72 (CON ST = 16, CON AR = 17, EXP ARdt = 21, EXP STdt = 18) Mean age_CONST_ = 71.1 ± 5.4 Mean age_CONAR_ = 70.5 ± 7.3 Mean age_EXPARdt_ = 71.9 ± 6.8 Mean age_EXPSTdt_ = 72.2 ± 5.9	ARdt = In addition to the aerobic and resistance training participants performed cognitive training on a PC (two visual discrimination tasks) STdt = In addition to stretching and toning training participants performed cognitive training on a PC (two visual discrimination tasks) PA training: 12 weeks: 2-time per week (60 min/trial) Cognitive training: 12 weeks: 1-time per week (60 min/trial)	AR = Aerobic exercises + passive computer lessons (excel, word) ST = Stretching exercises + passive computer lessons (excel, word) 12 weeks: 3-times per week (2x-physical exercise + 1x-computer lessons; 60 min/trial)	Gait speed - DT – ↑ for both CON and both EXP group
Sipilä et al. (2021)	*N =* 314 (CON=159, EX*P =* 155) Mean age_CON_ = 74.5 ± 3.7 Mean age_EXP_ = 74.4 ± 3.9	In addition to strength, balance, and aerobic training participants performed cognitive training on a PC (learning general computer skills) PA training: 1 year: 2-time per week (45 min/trial) + 2–3-times per week home based training (20–30 min/trial) Cognitive training: 1 year: 3–4-time per week (15–20 min/trial)	Strength, balance, and aerobic training 1 year: 2-time per week (45 min/trial) + 2–3-times per week home based training (20–30 min/trial)	Gait speed – ↑ for CON and EXP group

### Statistical Analysis

The meta-analyses were performed using Comprehensive Meta-analysis software (version 3.0; Biostat Inc., Englewood, NJ, USA). The mean differences and 95% confidence intervals (Cls) were calculated for the included studies. We applied the random-effects model of the meta-analysis in all comparisons to determine the effect of the motor-cognitive intervention on gait and balance. Due to the high heterogeneity of the measured variables, the effect sizes were reported in Hedges-‘g. To calculate each effect size we used reported mean value of selected parameter, their standard deviation and sample size of the included study. The following established criteria were used to interpret the magnitude of motor-cognitive intervention for gait and balance improvements: trivial (<0.20), small (0.21–0.60), moderate (0.61–1.20), large (1.21–2.00), very large (2.01–4.00) and extremely large (>4.00) changes (Hopkins et al., 2009; Fraser et al., 2017; Wongcharoen et al., 2017; Laatar et al., 2018). Heterogeneity across studies was assessed using the I^2^ statistics, which is a measure of inconsistency used to quantify between-study variability. A value of 25% is recommended to represent low statistical heterogeneity, 50% moderate and 75% high statistical heterogeneity (Higgins, 2003). In addition, the sensitivity analysis excluded studies with poor methodological quality, i.e., the study's PEDro score was 3 or less. The publication bias was assessed by examining the asymmetry of the funnel plots using Egger's test (Egger et al., 1997; Sterne et al., 2011). Significant publication bias was considered if the *p -* value was < 0.10.

## Results

The Egger's test was performed to provide statistical evidence of funnel plot asymmetry (see Supplementary Figures 2A–D). The results indicated publication bias for TUG only (*P* = 0.003).

### Study Selection

The initial search yielded 6,314 results. After duplicates were removed, 2,546 articles remained to be considered. After screening titles and abstracts, 890 records were excluded. Full-text reading of 351 articles revealed that 262 articles did not meet our inclusion criteria. We excluded 262 articles with the following reasons: no matched control group in the study (*n* = 44), unsuitable protocol (e.g., only intervention group, without control group) (*n* = 20), irrelevant outcomes (*n* = 32), unsuitable motor-cognitive interventions (e.g., additionally added cognitive tasks during interventions, incorporated cognitive task that was not part of exergame intervention) (*n* = 81), unsuitable measurement of gait (e.g., measures were performed on a treadmill) (*n* = 11), inadequate outcomes (*n* = 20), and unsuitable measurement of balance (e.g., measures were performed on a force plate) (*n* = 6) and not randomized controlled trials (*n* = 48). After excluding studies that included diseased older adults, there were 58 studies that we included in the quantitative synthesis. Details of the study selection process are presented in Supplementary Figure 1.

### Characteristics of Included Studies

Based on the quality assessment, 44 out of 58 studies were high quality, 11 of fair quality and only 3 of low quality (Table 2). The intervention characteristics, including the type of intervention, a description of the motor and cognitive components and the frequency and dose of training described in the included studies are summarized in Tables 3–5. To facilitate the review of included studies, we have subdivided all studies according to the type of training performed by the experimental group (sequential motor-cognitive training, simultaneous motor cognitive training with additional cognitive task and exergaming).

We included 7 studies with sequential motor-cognitive training (presented in Table 3). These studies included samples ranging from 14 to 147 participants (range of age 69.7–81.9 years). The most common training approach (*N* = 6) was a combination of aerobic/resistance/strength/balance training and performing cognitive tasks on the PC.

There were 19 studies with simultaneous motor-cognitive training with an additional cognitive task (presented in Table 4). Studies included samples ranging from 17 to 286 participants, with the age of participants in both groups (experimental and control group) varying from 64.7 to 85.3 years. The most common training approach used was a combination of balance training while simultaneously performing different cognitive tasks (the number of studies with that type of training is 9).

**Table 4:** Simultaneous motor-cognitive training with additional cognitive task.

**Study**	**Sample description**	**Experimental design and duration of trial period**	**Control design and duration of trial period**	**Outcomes and results**
Norouzi et al. (2019)	*N =* 60 (CON passive = 20, CON motor = 20, EXP = 20) Mean age_CONpassive_ = 68.1 ± 3.7 Mean age_CONmotor_ = 68.3 ± 4.1 Mean age_EXP_ = 68.5 ± 3.6	Resistance training wearing an isokinetic exercise device while simultaneously performing cognitive tasks (backward number counting, mental arithmetic, calculate the assignment to front, spelling particular names backwards, counting numbers backwards in intervals of 3 and 7, remembering words given in 300 ms intervals, remembering visual images, remembering shapes, remembering colors, differentiating between shapes, remembering the order of a word list). Four weeks: 3-times per week (60–80 min/trial)	CON passive = Passive group (only pre- and post-intervention assessment) CON motor = Resistance training with an isokinetic exercise device plus simultaneous motor training (skill training – throwing a bag, holding a bag, balancing the cup on the palm of the hand, holding a medicine ball in both hands)	BBS – ↑ for CON motor and EXP group
Eggenberger et al. (2015)	*N =* 47 (CON = 25, EXP = 22) Mean age_CON_ = 80.8 ± 4.7 Mean age_EXP_ = 78.5 ± 5.1	Treadmill walking while simultaneously performing cognitive tasks (verbal memory tasks) strength and balance exercises. 24 weeks: 2-times per week (60 min/trial)	Treadmill walking + strength and balance training 24 weeks: 2-times per week (60 min/trial)	Gait speed – ↑ for CON and EXP group Gait speed - DT – ↑ for CON and EXP group
Rezola-Pardo et al. (2019)	*N =* 85 (CON = 43, EXP = 42) Mean age_CON_ = 85.3 ± 7.1 Mean age_EXP_ = 84.9 ± 6.7	Strength and balance training while simultaneously performing cognitive tasks (different tasks to stimulate attention, executive functions and semantic memory). 12 weeks: 2-time per week (60 min/trial)	Strength and balance exercises 12 weeks: 2-time per week (60 min/trial)	Gait speed – ↑ for CON and EXP group, Gait speed - DT – ↑ for CON and EXP group TUG – ↑ for CON
Raichlen et al. (2020)	*N =* 53 (CON motor = 19, CONpassive = 14, EXP = 20) Mean age_CONmotor_ = 68.1 ± 3.9 Mean age_CONpassive_ = 69.3 ± 4.3 Mean age_EXP_ = 68.0 ± 4.7	Aerobic training while simultaneously performing cognitive tasks (memory, executive functions and processing speed exercises). 12 weeks: 3-time per week (20–35 min/trial)	CON motor = Aerobic exercise CON passive = Passive group (only pre- and post-intervention assessment) 12 weeks: 3-time per week (20–35 min/trial)	Gait speed -DT (no improvements)
Plummer-D'Amato et al. (2012)	*N =* 17 (CON = 7, EXP = 10) Mean age_CON_ = 76.7 ± 6.0 Mean age_EXP_ = 76.6 ± 5.6	Gait and balance training while simultaneously performing cognitive tasks (random number generation, word association, backward recitation, working memory). 4 weeks: 1-times per week (45 min/trial)	Gait, balance, and agility training 4 weeks: 1-time per week (45 min/trial)	Gait speed – ↑ for CON and EXP group, TUG – ↑ for CON and EXP group
Hiyamizu et al. (2012)	*N =* 36 (CON = 19, EXP = 17) Mean age_CON_ = 71.2 ± 4.4 Mean age_EXP_ = 72.9 ± 5.1	Strength and balance training while simultaneously performing cognitive tasks. 12 weeks: 2-times per week (60 min/trial)	Strength and balance training 12 weeks: 2-times per week (60 min/trial)	TUG (no improvement)
Wongcharoen et al. (2017)	*N =* 30 (CON motor = 15, EXP = 15) Mean age_CONmotor_ = 73.5 ± 5.9 Mean age_EXP_ = 71.9 ± 4.6	Balance training while simultaneously performing cognitive tasks (visuospatial skills, executive functions, attention, and working memory exercises). 4 weeks: 3-times per week (60 min/trial)	CON motor = Balance training 4 weeks: 3-times per week (60 min/trial)	Gait speed - ↑ for CON motor and EXP group. Gait speed - DT – - ↑ for CON motor and EXP group.
Nematollahi et al. (2016)	*N =* 29 (CON = 14, EXP = 15) Mean age_CON_ = 67.7 ± 5.0 Mean age_EXP_ = 64.7 ± 5.0	Balance training while simultaneously performing cognitive tasks (adding numbers, counting backwards by 3s during narrow-base walking, naming the opposite direction of their actions). 4 weeks: 3-times per week (60 min/trial)	Balance training 4 weeks: 3-times per week (60 min/trial)	Gait speed (no improvement)
Gregory et al. (2016)	*N =* 44 (CON = 21, EXP = 23) Mean age_CON_ = 74.5 ± 7.0 Mean age_EXP_ = 72.6 ± 7.4	Aerobic, strength, balance, and flexibility training while simultaneously performing cognitive tasks (beginner-level square stepping exercise). 26 weeks: 2–3-times per week (60–75 min/trial)	Aerobic, strength, balance, and flexibility training 26 weeks: 2–3-times per week (60–75 min/trial)	Gait speed – DT - ↑ for EXP group
Nishiguchi et al. (2015)	*N =* 48 (CON = 24, EXP = 24) Mean age_CON_ = 73.5 ± 5.6 Mean age_EXP_ = 73.0 ± 4.8	Walking training while simultaneously performing different cognitive tasks (different verbal fluency tasks) 12 weeks: 1-time per week (90 min/trial)	Passive group (only pre- and post-intervention assessment)	Gait speed - ↑ EXP group TUG (no improvement)
Yamada et al. (2011a)	*N =* 53 (CON *=* 27, EXP = 26) Mean age_CON_= 81.2 ± 7.6 Mean age_EXP_ = 80.3 ± 5.4	Stepping training while simultaneously performing cognitive tasks (different verbal fluency tasks) 24 weeks: 1-time per week (50 min/trial)	Stepping training 24 weeks: 1-time per week (50 min/trial)	Gait speed - DT – ↑ EXP group Gait speed and TUG (no improvement)
Yamada et al. (2011b)	*N =* 93 (CON = 45, EXP = 48) Mean age_CON_ = 82.9 ± 5.5 Mean age_EXP_ = 83.0 ± 6.7	Stretching, strength and agility training (seated) + seated stepping exercise while simultaneously performing cognitive tasks (verbal fluency tasks such as listing wirds within a category at a self selected speed) 24 weeks: 2-time per week (20 min/trial)	Passive group (only pre- and post-intervention assessment)	Gait speed - DT – ↑ EXP group Gait speed and TUG (no improvement)
Theill et al. (2013)	*N =* 41 (CON passive = 21, EXP = 18) Mean age_CONpassive_ = 70.9 ± 4.8 Mean age_EXP_ = 72.4 ± 4.2	Treadmill walking while simultaneously performing cognitive tasks (computer based tasks). 10 weeks: 2-times per week (40 min/trial)	CON passive = Passive group (participants were tested before and after intervention) 10 weeks: 2-times per week (15 min/trial)	Gait speed – ↑ for CON and EXP group, Gait speed - DT – ↑ for CON and EXP group,
Salazar-González et al. (2015)	*N =* 286 (CON = 143, EXP = 143) Mean age_CON_ = 74.0 ± 6.3 Mean age_EXP_ = 71.0 ± 5.7	Treadmill walking while simultaneously performing cognitive tasks. 12 weeks: 3-times per week (60 min/trial)	Passive group (participants were tested before and after intervention)	Gait speed – ↑ for EXP group
Jehu et al. (2017)	*N =* 26 (CONpassive= 12, CONmotor = 15, EXP = 14) Mean age_CONpassive_ = 66.3 ± 4.4 Mean age_CON_ _motor_ = 70.2 ± 3.1 Mean age_EXP_ = 68.7 ± 5.5	Balance training while simultaneously performing cognitive tasks. 12 weeks: 3-times per week (60 min/trial)	CONpassive = Passive group (only pre- and post-intervention assessment) CONmotor = Balance and mobility training 12 weeks: 3-times per week (60 min/trial)	TUG – ↑ for CON motor and EXP group
Medeiros et al. (2018)	*N =* 71 (CON = 36, EXP = 35) Mean age_CON_ = 68.1 ± 6.4 Mean age_EXP_ = 67.8 ± 8.6	Aerobic, flexibility, strength, and balance training while simultaneously performing cognitive tasks. 12 weeks, 3-times per week (50 min/trial)	Aerobic, flexibility, strength, and balance training 12 weeks: 3-times per week (50 min/trial)	TUG (no improvement)
Bischoff et al. (2020)	*N =* 24 (CON = 2, EXP = 5) Mean age_CON_ *=* 83.8 ± 5.7 Mean age_EXP_ = 83.6 ± 7.3	Balance, coordination, aerobic and strength training while simultaneously performing cognitive tasks. 16 weeks: 2-times per week (45 - 60 min/trial)	Balance, coordination, aerobic and strength training 16 weeks: 2-times per week (45–60 min/trial)	Gait speed
Jardim et al. (2021)	*N =* 72 (CON *=* 31, EXP = 41) Mean age_CON_ = 83.8 ± 5.7 Mean age_EXP_ = 83.6 ± 7.3	Aerobic, resistance, and stretching training while simultaneously performing cognitive tasks. 12 weeks: 2-times per week (75 min/trial)	Passive group (only pre- and post-intervention assessment)	Gait speed – ↑ for EXP group TUG – ↑ for EXP group
Falbo et al. (2016)	*N =* 36 (CON = 16, EXP = 20) Mean age_CON_ = 73.7 ± 4.5 Mean age_EXP_ = 71.5 ± 6.7	Aerobic, strength, agility, balance and stretching training while simultaneously performing cognitive tasks relying on executive functions. 12 weeks: 2-time per week (60 min/trial)	Aerobic, strength, agility, balance and stretching training while simultaneously performing physical dual task exercise.	Gait speed -DT

We included 33 studies that performed exergaming (Table 5). Studies included samples ranging from 9 to 153 participants. The age range of participants in both groups (experimental and control) was between 65.2 and 86.9 years. The training approach in the studies included different types of interventions using PC and consoles with game controls, e.g., Nintendo Wii and Xbox Kinect, as one form of exergaming training offered to the participants.

**Table 5:** Exergaming.

**Study**	**Sample description**	**Experimental design and duration of trial period**	**Control design and duration of trial period**	**Outcomes and results**
Eggenberger et al. (2015)	*N =* 49 (CON = 25, EXP = 24) Mean age_CON =_80.8 ± 4.7 Mean age_EXP_ = 77.3 ± 6.3	Video game dancing + strength and balance exercises 24 weeks: 2-times per week (60 min/trial)	Treadmill walking + strength and balance training 24 weeks: 2-times per week (60 min/trial)	Gait speed – ↑ for CON and EXP group Gait speed - DT – ↑ for CON and EXP group
Morat et al. (2019)	*N =* 30 (CON = 15, EXP = 15) Mean age_CON_ = 71.1 ± 5.2 Mean age_EXP_ = 69.7 ± 6.2	Volitional stepping exergame on the Dividat Senso device 8 weeks: 3-times per week (40 min/trial)	Passive group (only pre- and post-intervention assessment)	TUG – ↑ for EXP group
Phirom et al. (2020)	*N =* 39 (CON = 19, EXP = 20) Mean age_CON_ = 71.1 ± 5.2 Mean age_EXP_ = 69.7 ± 6.2	Xbox 360 Kinect – stepping on different targets and in different directions, and balance training. 12 weeks: 3-times per week (60 min/trial)	Passive group (only pre- and post-intervention assessment)	TUG – ↑ for EXP group
Kao et al. (2018)	*N =* 62 (CON = 31, EXP = 31) Mean age_CON_ = 72.3 ± / Mean age_EXP_ = 73.5 ± /	Hot Plus interactive health service system – psychomotor skills training 8 weeks: 3-times per week (30 min/trial)	Active control group by use of a tablet computer for the passive information activity.	Gait speed (no improvement)
Karahan et al. (2015)	*N =* 90 (CON = 42, EXP = 48) Mean age_CON_ = 71.5 ± 4.7 Mean age_EXP_ = 71.3 ± 6.1	Xbox 360 Kinect 6 weeks: 5-times per week (30 min/trial)	Home-based balance training 6 weeks: 5-times per week (30 min/trial)	BBS – ↑ for CON and EXP group TUG – ↑ for EXP group
Pichierri et al. (2012)	*N =* 21 (CON = 10, EXP = 11) Mean age_CON_ = 85.6 ± 4.2 Mean age_EXP_ = 86.9 ± 5.1	Resistance and balance training + video game dancing 12 weeks: PA: 2-times per week (40 min/trial) + video game dancing: 2-times per week (10–15 min/trial)	Resistance and balance training 12 weeks: PA: 2-times per week (40 min/trial)	Gait speed (no improvement) Gait speed - DT – ↑ for CON and EXP group,
Schwenk et al. (2014)	*N =* 33 (CON *=* 16, EXP = 17) Mean age_CON =_84.9 ± 6.6 Mean age_EXP_ = 84.3 ± 7.3	Balance training including weight shifting and virtual obstacle crossing tasks with visual/auditory real-time joint movement feedback using wearable sensors. 4 weeks: 2-times per week (45 min/trial)	Passive group (only pre- and post-intervention assessment)	Gait speed – ↑ EXP group TUG – ↑ for EXP group
Schoene et al. (2013)	*N =* 32 (CON *=* 17, EXP = 15) Mean age_CON =_78.4 ± 4.5 Mean age_EXP_ = 77.5 ± 4.5	Step training using a videogame technology (DDR) 8 weeks: 2–3-times per week (15–20 min/trial)	Passive group (only pre- and post-intervention assessment)	TUG (no improvement)
Bieryla and Dold (2013)	*N =* 9 (CON *=* 5, EXP = 4) Mean age_CON =_80.5 ± 7.8 Mean age_EXP_ = 82.5 ± 1.6	Wii Balance Board with Wii Fit training 3 weeks: 3-times per week (30 min/trial)	Passive group (only pre- and post-intervention assessment)	TUG (no improvement)
Bieryla (2016)	*N =* 12 (CON *=* 7, EXP = 5) Mean age_CON =_82.6 ± 6.9 Mean age_EXP_ = 82.0 ± 2.4	Xbox Kinect training to improve balance. 3 weeks: 3-times per week (30 min/trial)	Passive group (only pre- and post-intervention assessment)	BBS – ↑ for EXP group TUG (no improvement)
Chao et al. (2015)	*N =* 32 (CON *=* 16, EXP = 16) Mean age_CON =_83.7 ± 8.0 Mean age_EXP_ = 86.6 ± 4.2	Wii Fit training 4 weeks: 2-times per week (30 min/trial)	Health educational session 4 weeks: 1-times per week (30 min/trial)	BBS – ↑ for EXP group TUG – ↑ for EXP group Gait speed (6MWT) (no improvement)
Sápi et al. (2019)	*N =* 53 (CONmotor= 23, CONpassive = 22, EXP = 30) Mean age_CON_ _motor_ = 69.7 ± 4.7 Mean age_CON_ _passiver_ = 67.2 ± 5.6 Mean age_EXP_ = 69.1 ± 4.2	Kinect balance training 6 weeks: 3-times per week (30 min/trial)	CONmotor = Conventional balance training CONpassive= Passive group (only pre- and post-intervention assessment) 6 weeks: 3-times per week (30 min/trial)	TUG – ↑ for CON motor and EXP group
Sato et al. (2015)	*N =* 54 (CON *=* 26, EXP = 28) Mean age_CON =_68.5 ± 5.5 Mean age_EXP_ = 70.1 ± 5.3	Kinect training 12 weeks: 1-2-times per week (30 min/trial)	Passive group (only pre- and post-intervention assessment)	Gait speed BBS – ↑ for EXP group
Schättin et al. (2016)	*N =* 27 (CON *=* 14, EXP = 13) Mean age_CON =_72.2 ± / Mean age_EXP_ = 73.0 ± /	Exergame training 8 weeks: 3-times per week (30 min/trial)	Conventional balance training 8 weeks: 3-times per week (30 min/trial)	Gait speed – ↑ CON group Gait speed - DT – ↑ for EXP group
Sadeghi et al. (2021)	*N =* 44 (CON motor = 14, CON passive = 15, EXP = 15) Mean age_CONmotor_ = 70.4 ± 4.3 Mean age_CONpassive_ = 72.2 ± 7.2 Mean age_EXP_ = 74.1 ± 7.0	Virtual reality balance training 8 weeks: 3-times per week (40 min/trial)	CON motor = Traditional balance training CON passive = Passive group (only pre- and post-intervention assessment) 8 weeks: 3-times per week (40 min/trial)	Gait speed – ↑ CON motor and EXP group TUG – ↑ CON motor and EXP group
Gallardo-Meza et al. (2022)	*N =* 72 (CON *=* 37, EXP = 35) Mean age_CON =_69.2 ± 3.7 Mean age_EXP_ = 74.1 ± 7.0	Nintendo Wii training (Wii Fit Plus; Wii Balanceboard; Wii Nunchuk) 4 weeks: exergame:2-times per week (40 min/trial) + Recreational physical activity training: 1-time per week (40 min/trial)	Recreational physical activity training 4 weeks: 3-times per week (40 min/trial)	TUG – ↑ for EXP group
Lai et al. (2013)	*N =* 30 (CON *=* 15, EXP = 15) Mean age_CON =_74.8 ± 4.7 Mean age_EXP_ = 70.6 ± 3.5	Interactive video game training (The Xavix Measured Step System) 6 weeks: 3-times per week (30 min/trial)	Passive group (only pre- and post-intervention assessment)	TUG – ↑ for EXP group BBS – ↑ for EXP group
Maillot et al. (2012)	*N =* 32 (CON *=* 16, EXP = 16) Mean age_CON =_73.5 ± 3.0 Mean age_EXP_ = 73.5 ± 4.1	Nintendo Wii training (Wii Balanceboard; Wii Nunchuk) 12 weeks: 2-times per week (60 min/trial)	Passive group (only pre- and post-intervention assessment)	Gait speed ↑ for EXP group TUG ↑ for EXP group
Gschwind et al. (2015)	*N =* 153 (CON *=* 75, EXP = 78) Mean age_CON =_74.7 ± 6.0 Mean age_EXP_ = 74.7 ± 6.7	Balance exergame training (iStoppFalls) + strength training 16 weeks: exergame: 2-times per week (60 min/trial) + strength training: 3-times per week (20 min/trial)	Passive group (only pre- and post-intervention assessment)	Gait speed (10 MWT) -DT; TUG (no improvement)
Gschwind et al. (2015)	*N =* 124 (CON *=* 61, EXP_kin =_24, EXPsmt=39) Mean age_CON =_80.2 ± 6.5 Mean age_EXP−kin =_ 80.1 ± 6.3 Mean age_EXP−smt_ = 82.5 ± 7.0	Home-based interventions of Kinect balance training or Step-mat-training (SMT) exergame Kinect: 16 weeks: exergame: 2-times per week (60 min/trial) + strength training: 3-times per week (20 min/trial) SMT: 16 weeks: exergame: 3-times per week (20 min/trial)	Passive group (only pre- and post-intervention assessment)	TUG (no improvement)
Rendon et al. (2012)	*N =* 34 (CON *=* 18, EXP = 16) Mean age_CON =_83.3 ± 6.2 Mean age_EXP_ = 85.7 ± 4.3	Wii Fit balance training 6 weeks: 3-times per week (35–45 min/trial)	Passive group (only pre- and post-intervention assessment)	TUG (8 feet up and go) – ↑ for EXP group
Franco et al. (2012)	*N =* 32 (CON motor = 11, CON passive = 10, EXP = 11) Mean age_CONmotor_ = 77.9 ± 6.9 Mean age_CONpassive_ = 76.9 ± 6.3 Mean age_EXP_ = 79.8 ± 4.7	Wii Fit balance group 3 weeks: 2-times per week (10–15 min/trial)	CON motor = Matter of balance group CON passive = Passive group (only pre- and post-intervention assessment) 3 weeks: 2-times per week (30–45 min/trial)	BBS – ↑ for CON motor and EXP group
Pluchino et al. (2012)	*N =* 26 (CON *=* 14, EXP = 12) Mean age_CON =_76.0 ± 7.7 Mean age_EXP_ = 70.7 ± 8.5	Video game balance board training 8 weeks: 2-times per week (60 min/trial)	Balance training program 8 weeks: 2-times per week (60 min/trial)	TUG (no improvement)
Jorgensen et al. (2013)	*N =* 58 (CON *=* 30, EXP = 28) Mean age_CON =_73.7 ± 6.1 Mean age_EXP_ = 75.9 ± 5.7	Biofeedback-based Nintendo Wii training 10 weeks: 2-times per week (30-40 min/trial)	Ethylene vinyl acetate copolymer insoles	TUG – ↑ for EXP group
Park et al. (2015)	*N =* 24 (CON *=* 12, EXP = 12) Mean age_CON =_65.2 ± 7.9 Mean age_EXP_ = 66.5 ± 8.1	Virtual reality training (Wii Fit balance exercise) 8 weeks: 3-times per week (30 min/trial)	Ball game training 8 weeks: 3-times per week (30 min/trial)	TUG – ↑ for CON and EXP group
Kwok and Pua (2016)	*N =* 80 (CON *=* 40, EXP = 40) Mean age_CON =_70.5 ± 6.7 Mean age_EXP_ = 69.8 ± 7.5	Wii exercise program 12 weeks: 1-times per week (60 min/trial)	Standard Gym-based exercise 12 weeks: 1-times per week (60 min/trial)	TUG – ↑ for CON and EXP group Gait speed (6MWD) – ↑ for CON and EXP group
Yesilyaprak et al. (2016)	*N =* 18 (CON *=* 11, EXP = 7) Mean age_CON =_70.1 ± 4.0 Mean age_EXP_ = 73.1 ± 4.5	Virtual reality balance training (BTS NIRVANA VR Interactive System) 6 weeks: 3-times per week (45–60 min/trial)	Conventional balance training 6 weeks: 3-times per week (45-60 min/trial)	BBS – ↑ for CON and EXP group TUG – ↑ for CON and EXP group
Eggenberger et al. (2016)	*N =* 33 (CON *=* 14, EXP = 19) Mean age_CON =_77.8 ± 7.4 Mean age_EXP_ = 72.8 ± 5.9	Interactive cognitive-motor video game dancing 8 weeks: 3-times per week (30 min/trial)	Balance and stretching training 8 weeks: 3-times per week (30 min/trial)	Gait speed (4MWT) (no improvement)
Padala et al. (2012)	*N =* 22 (CON *=* 11, EXP = 11) Mean age_CON =_81.6 ± 5.2 Mean age_EXP_ = 79.3 ± 9.8	Wii Fit training 8 weeks: 5-times per week (30 min/trial)	Walking training 8 weeks: 5-times per week (30 min/trial)	BBS – ↑ for CON and EXP group TUG – ↑ CON group
Lee et al. (2018)	*N =* 40 (CON *=* 21, EXP = 19) Mean age_CON =_75.7 ± 4.9 Mean age_EXP_ = 76.2 ± 4.6	Virtual reality training program 6 weeks: 2-times per week (60 min/trial)	Passive group (only pre- and post-intervention assessment)	BBS – ↑ for EXP group TUG – ↑ for EXP group
Nagano et al. (2016)	*N =* 39 (CON *=* 19, EXP = 20) Mean age_CON =_72.0 ± 5.0 Mean age_EXP_ = 72.0 ± 5.0	Stepping mat exergame 12 weeks: 2-times per week (15 min/trial)	Passive group (only pre- and post-intervention assessment)	Gait speed (10 MWT) TUG – ↑ for EXP group
Yoo et al. (2013)	*N =* 21 (CON *=* 11, EXP = 10) Mean age_CON =_75.6 ± 5.6 Mean age_EXP_ = 72.9 ± 3.4	Augmented reality-based Otago exercise 12 weeks: 3-times per week (60 min/trial)	Otago exercise group 12 weeks: 3-times per week (60 min/trial)	Gait speed – ↑ for CON and EXP group BBS – ↑ for CON and EXP group
Lee et al. (2015)	*N =* 54 (CON *=* 28, EXP = 26) Mean age_CON =_67.7 ± 4.3 Mean age_EXP_ = 68.8 ± 4.6	Individualized feedback-based virtual reality exercise 8 weeks: 3-times per week (60 min/trial)	Postural, balance, functional, lower body coordination, and lower body strength exercises 8 weeks: 3-times per week (60 min/trial)	8FUGT – ↑ for CON and EXP group

### Meta-Analysis Outcomes (Domain-Specific Efficacy)

#### Gait Speed Under a Single-Task Condition

Forty-one studies (41 ESs) were included to assess the effect of motor-cognitive intervention on gait speed under a single-task condition. The results showed that the motor-cognitive intervention has a small positive effect on gait speed under a single-task condition (ES = 0.34, 95% Cl 0.12 to 0.57, *P* = 0.003). The effect of the intervention was moderated by the control group (Q = 6.203, *P* = 0.013); i.e., passive (ES = 0.941, 95% Cl 0.36 to 1.52, *P* = 0.001) vs. active (ES = 0.153, 95% Cl −0.07 to 0.38, *P* = 0.180). The type of intervention did not bring significant differences where additional, incorporated, and sequential intervention had the same effect on gait speed under a single task (Q = 0.668; *P* = 0.716). Even after excluding the study (Eggenberger et al., 2016) that had a low PEDro score (PEDro score = 3), the results showed a small positive effect of the motor-cognitive intervention on a gait speed under a single-task condition (ES = 0.35, 95% Cl 0.12 to 0.58, *P* = 0.003) and the effect of the intervention was moderated by the control group (*Q* = 6.067, *P* = 0.014); i.e., passive (ES = 0.941, 95% Cl 0.36 to 1.52, *P* = 0.001) vs. active (ES = 0.159, 95% Cl −0.07 to 0.39, *P* = 0.177). Moreover, once again the type of intervention did not bring significant difference on a gait speed under a single task (*Q* = 0.799; *P* = 0.671).

#### Gait Speed Under a Dual-Task Condition

Twenty studies (20 ESs) were included to assess the effect of motor-cognitive intervention on gait speed under a dual-task condition. The results showed that the motor-cognitive intervention has no significant effect on gait speed under a dual-task condition (ES = 0.22, 95% Cl −0.01 to 0.44, *P* = 0.063). There was no significant difference between different control groups (*Q* = 0.003; *P* = 0.957) nor the type of intervention (Q = 0.213; *P* = 0.899).

#### Timed Up and Go Test

Forty-one studies (41 ESs) were included to assess the effect of motor-cognitive intervention on TUG test. The results showed that the motor-cognitive intervention has a small positive effect on TUG (ES = 0.42, 95% Cl 0.21 to 0.63, *P* < 0.001). The effect of the intervention was moderated by the control group (Q = 4.92; *P* = 0.027); i.e., passive (ES = 0.73, 95% Cl 0.30 to 1.15, *P* = 0.001) vs. active (ES = 0.20, 95% Cl 0.03 to 0.38, *P* = 0.020), but not by the type of training (*Q* = 5.51; *P* = 0.064). After conducting sensitivity analysis by excluding two studies with low PEDro score (Bieryla and Dold, 2013; Sápi et al., 2019), the results still showed a small positive effect of the motor-cognitive intervention on TUG results (ES = 0.35, 95% Cl 0.15 to 0.55, *P* = 0.001) and the effect of the intervention was still moderated by the control group (*Q* = 4.280, *P* = 0.039); i.e., passive (ES = 0.619, 95% Cl 0.22 to 1.02, *P* = 0.003) vs. active (ES = 0.160, 95% Cl 0.001 to 0.320, *P* = 0.048). There was no significant difference between different types of intervention after excluding low quality studies.

#### Berg Balance Scale

Eleven studies (11 ESs) were included to assess the effect of motor-cognitive intervention on BBS score. The results showed that the motor-cognitive intervention has a small positive effect on BBS (ES = 0.59, 95% Cl 0.39 to 0.79, *P* < 0.001). There was no difference between active or passive control group (*Q* = 0.397; *P* = 0.529) and no difference between the type of intervention (*Q* = 0.299; *P* = 0.585).

## Discussion

The aim of the present systematic review and meta-analysis was to investigate whether motor-cognitive interventions can have a positive impact on selected gait and balance parameters in older adults. We contrasted the effects of passive and active control groups as well as three different types of motor-cognitive training. We focused on motor-cognitive interventions such as dual-task training and included studies with healthy older adults. Because of high heterogeneity in the studies performed in diseased older adults, we excluded them and performed the analysis only on healthy older people. To increase sensitivity, we additionally excluded three studies from the analysis that were of poor quality according to the PEDro assessment.

Overall, we found evidence that motor-cognitive interventions can improve gait speed under single-task condition, and measures of functional balance, but they have no significant effects on dual-task walking outcomes. However, motor-cognitive intervention does not necessarily have a better effect on gait speed under single-task improvement than active control group, which in this case is conventional motor training. On the other hand, there was a small but significant effect in favor of motor-cognitive interventions compared with other conventional motor interventions for TUG. Finally, we found that most studies conducted motor-cognitive training with additional cognitive tasks (*n* = 53), fewer studies conducted exergaming training (48). The least research has been conducted with sequential motor-cognitive training (*n* = 8). However, the studies differed in terms of intervention protocol, frequency, dosage of training, and sample size. Therefore, considerable heterogeneity was found among the included studies in terms of the methodology used. In the next sections we discuss the results per outcomes of interest in some more detail.

### Gait Speed Under a Single-Task Condition

Our meta-analysis showed that motor-cognitive intervention can improve gait speed under single-task conditions. There was a small but significant effect that suggests that motor-cognitive interventions for this specific gait parameter may be beneficial for healthy older adults. However, further analysis showed that such interventions are no more effective than other conventional interventions in improving gait speed under a single-task conditions and that the type of motor-cognitive intervention is not a moderating factor for a positive effect.

Although walking was considered a fairly simple task until recently, it requires a large amount of higher-level cognitive input (Mirelman et al., 2018). For this reason, we hypothesized that motor-cognitive interventions would be more beneficial compared to motor training alone. The fact that motor-cognitive training does not contribute more to gait improvement in the active comparison groups than conventional forms of motor training alone is an important point to discuss. The finding is in line with one other systematic review (Gavelin et al., 2021) showing that the addition of cognitive training to physical exercise does not reduce physical efficacy of the training, and exergaming was only superior to passive control for both physical and cognitive outcomes. However, this review also found that motor-cognitive training is likely to be most effective for cognition.

The effect size of the overall improvement in gait speed under a single-task conditions was small, with high statistical heterogeneity between studies. Because of the great heterogeneity in the methods and measurements of the studies, more studies within each subgroup would be needed to draw definitive conclusions. When planning future motor-cognitive interventions, overall effectiveness is important and a deeper understanding of causation is needed (e.g., type and design of intervention, quality of research conducted). There were 27 studies in which motor-cognitive intervention had a positive effect and 14 studies in which motor-cognitive intervention had a negative effect. Among the studies with the positive effect, the highest effect of intervention had the study by Sadeghi et al. (2021) (quality score = 6/11, see Table 2). The experimental design of this study involved a motor-cognitive intervention with visual context displayed on a PC, a confirmed human-computer interaction with tasks performed dynamically. The effect of an intervention study conducted by Jardim et al. (2021) (quality score = 6/11), in which participants performed aerobic, resistance, and stretching training while simultaneously solving cognitive tasks, was similarly high. The third largest effect was in the study by Pothier et al. (2018) (quality score = 4/11), where participants in addition to the aerobic and resistance training, performed cognitive training on a PC. Taken together, analysis of studies with larger effect sizes did not identify any pure trends that could currently provide an answer to the most effective designs of motor-cognitive interventions.

Finally, an additional analysis was performed excluding the study by Eggenberger et al. (2016) due to poor quality (PEDro score ≤ 3). The exclusion of the study did not affect the final conclusion of the results, as the additional calculations only confirmed the results reported above.

### Gait Speed Under a Dual-Task Condition

We included twenty studies in the meta-analysis to evaluate the effect of a motor-cognitive intervention on gait speed under dual-task conditions, which yielded a non-significant effect. Regarding the quality (assessed by the PEDro scale) of the included studies, 6 studies were of “good quality” and 5 studies were of “fair quality”. Heterogeneity was moderate (82%), and the dual-task assessment methods varied considerably. When interpreting our non-significant results on gait speed under dual-task conditions, it should also be considered that dual-task walking used different cognitive tasks and were combined into one effect size (e.g., walking with n-back task, verbal fluency task, backward couniting, Go/No Go task). Therefore, future studies should investigate this effect considering different subcategories of cognition as a secondary task.

### Timed Up and Go Test Outcomes

We found that motor-cognitive intervention has a small positive effect on TUG performance for healthy individuals. Moreover, our analysis showed that motor-cognitive intervention is more effective than other conventional interventions in improving the TUG test, but the type of motor-cognitive intervention is not a moderating factor for a positive effect.

The overall effect size was small, with the majority of included studies showing a positive effect of motor-cognitive intervention on TUG test. We included 41 studies, of which 33 had a positive effect size and 8 had a negative effect size. The three interventions related to TUG performance with high effect sizes differed in the type of motor-cognitive intervention as well as in the study quality ratings; one study conducted motor-cognitive training with an additional cognitive task (Jardim et al., 2021; quality score = 6/11). The experimental design included the simultaneous performance of aerobic, resistance, and stretching training, in addition to the performance of various cognitive tasks. Two studies conducted motor-cognitive training with an incorporated cognitive task (Sápi et al., 2019, quality score = 3/11, Kinect balance training; Sadeghi et al., 2021, quality score = 6/11, balance training in virtual reality). On the other hand, the highest negative effect was found by Medeiros et al. (2018) (quality score = 7/11), but it was still only a small negative effect. In the latter study, participants in the experimental group performed a combination of aerobic, flexibility, strength, and balance training while completing a cognitive task. The authors explained the negative effect by the type of the sample (participants had exercised before the intervention) and the relatively short duration of the intervention (12 weeks). Similar to gait speed under a single-task condition, no clear trend for the most effective design of motor-cognitive intervention can be derived for the TUG test.

In addition, the type of control group (passive vs. active) moderated the effects of motor-cognitive interventions, suggesting that motor-cognitive interventions are better able to improve multicomponent tasks of dynamic balance and mobility function as measured by the TUG. Indeed, both the passive and active control groups had a significant effect on the results. The effect of the passive control group was moderate, while the effect of the active control group was smaller, as expected (ES = small), but still statistically significant. Since TUG is a multicomponent test that examines balance, gait speed, and functional ability (Beauchet et al., 2011), it achieves higher ecological validity compared with less complex straight-line walking without an additional task. Motor-cognitive interventions could therefore be a promising strategy to improve dynamic balance and mobility in older adults. When performing a sub-analysis of different types of motor-cognitive interventions (although there was only a non-significant trend with *P* = 0.064), both the additional and incorporated interventions had a positive effect on TUG, but the sequential intervention did not. One possible explanation is that performing motor and cognitive tasks at different times (sequential motor-cognitive training) may not be as stimulating for improving complex movement tasks as performing these tasks simultaneously. In addition, Prosperini et al. (2021) conducted a meta-analysis in which they found that exergaming interventions can have a positive impact on the balance of people with neurological disorders. This study (Prosperini et al., 2021) was performed on a group of diseased older adults, and we cannot directly confirm our findings with the above-mentioned study, but related results may help us draw meaningful conclusions on this topic. That is, a motor-cognitive intervention with incorporated cognitive task or exergaming has already been shown to have a positive effect on the selected population.

In addition, our results were also confirmed by the exclusion of two studies that were considered to be of poor quality by the PEDro assessment (Bieryla and Dold, 2013; Sápi et al., 2019). Reanalysis confirmed a small but positive effect of motor-cognitive intervention on TUG scores and the effect of the intervention was still moderated by the control group.

### Berg Balance Scale Outcomes

The 11 studies included in our meta-analysis showed a positive but small effect on Berg Balance Scale scores (BBS). Thus, motor-cognitive intervention may be beneficial for healthy older adults while improving BBS score, but no differences were found between control groups or type of intervention. All included studies had a positive effect on BBS. The highest effect of the intervention was found in the studies by Karahan et al. (2015) (quality score = 7/11) and Norouzi et al. (2019) (quality score = 8/11). Participants in the experimental group in the study by Karahan et al. (2015) performed exergaming training, where they exercised on the Xbox 360 Kinect. The control group participated in balance training at home. On the other hand, participants in the experimental group in the study by Norouzi et al. (2019) performed resistance training using an isokinetic training device while performing cognitive tasks. Considering that BBS evaluates static and dynamic balance, the possible explanation for the results in favor of the control group could be the implementation of conventional physical therapy, that is motor training. The conventional motor training may have a better effect on balance parameters (BBS) than walking or playing video games. In addition, the existing literature summarizing the effect of simultaneous motor-cognitive training with incorporated cognitive task is inconsistent; Howes et al. (2017) and Pacheco et al. (2020) concluded that exergaming can improve static balance measured with BBS, whereas Chen et al. (2021) did not reach this conclusion.

### Limitations and Future Directions

There are also some limitations as well as future directions that should be mentioned. First, despite a high heterogeneity among motor-cognitive approaches in terms of the selected cognitive or motor task, duration, and frequency, we pooled and summarized the data for the meta-analysis. Second, our meta-analysis included participants with a mean age of 60 years, allowing for the possibility that some individuals in the studies were younger than this age. Third, the results of publication bias indicated the presence of bias in TUG and BBS outcomes. Future studies should focus on other aspects of functional mobility and examine the effectiveness of such motor-cognitive interventions on activities of daily living, such as navigating parks and grocery stores, ability to drive, and others. Finally, the current scarcity of literature on motor-cognitive interventions in specific disease populations may open new avenues of discussion for the implementation of such training. For example, technology-driven exergames with forms of extended reality (XR) that combine real and virtual environments and relate to human-machine interactions generated by computers and wearable technologies will provide services for remote monitoring, training, and telerehabilitation (Meulenberg et al., 2022). The nature of engagement in XR allows training and/or rehabilitation exercises to feel similar to physically performed actions, while seemingly being more engaging, motivating, and stimulating than conventional practice.

## Conclusion

This systematic review and meta-analysis shows that conventional motor-cognitive interventions and technology-based exergames can improve performance-based measures of functional mobility in older adults. Our results show that motor-cognitive interventions can be effective, particularly in the multicomponent daily tasks that older adults encounter and that resemble the TUG test-mobility tasks of transferring from sitting to standing and walking, as well as balance tasks during walking, stopping, and turning. Because of substantial heterogeneity and the current limited availability of different types of interventions, conclusions should be drawn with caution. Further dose-response studies should be conducted to determine the appropriate training dose for this specific population. New insights into training design, as well as recent advances in immersive and wearable technology, offer a new perspective for implementing motor-cognitive interventions as more comprehensive training tools to improve functional mobility in the elderly and increase their effectiveness.

## Data Availability Statement

The original contributions presented in the study are included in the article/Supplementary Material, further inquiries can be directed to the corresponding author/s.

## Author Contributions

KT and UM designed the research. KT, LŠ, and AP performed the data extraction and performed the meta-analysis. KT, EB, and UM drafted the manuscript. KT, LŠ, EB, AP, and UM edited and revised the manuscript. All authors approved the final version of the manuscript.

## Funding

The authors acknowledge the financial support from the Slovenian Research Agency (research core funding No. P5-0381). This study was also supported by the European Union's Horizon 2020 research and innovation programme under grant agreement no. 952401 (TwinBrain – TWINning the BRAIN with machine learning for neuro-muscular efficiency).

## Conflict of Interest

The authors declare that the research was conducted in the absence of any commercial or financial relationships that could be construed as a potential conflict of interest.

## Publisher's Note

All claims expressed in this article are solely those of the authors and do not necessarily represent those of their affiliated organizations, or those of the publisher, the editors and the reviewers. Any product that may be evaluated in this article, or claim that may be made by its manufacturer, is not guaranteed or endorsed by the publisher.

## Footnotes

^1^We found a high degree of heterogeneity within the groups diagnosed with a particular deficit, so we decided to exclude all studies that included diseased individuals from further analysis. However, we have left a summary of all studies included in the original analysis in Supplementary Tables 1–4.
