# Supplementary material for: Impact of Motor-Cognitive Interventions on Selected Gait and Balance Outcomes in Older Adults: A Systematic Review and Meta-Analysis of Randomized Controlled Trials
Source: Front Psychol. 2022 Jun 16;13:837710. doi: 10.3389/fpsyg.2022.837710 (PMC9245546; doi:10.3389/fpsyg.2022.837710)
Supplement: Supplementary file 1 [file Table_1.docx]

Table 1

Quality assessment of included studies with diseased older adults according to PEDro scale

| Study | Quality criteria | | | | | | | | | | | Quality Score |
| --- | --- | --- | --- | --- | --- | --- | --- | --- | --- | --- | --- | --- |
|  | 1 | 2 | 3 | 4 | 5 | 6 | 7 | 8 | 9 | 10 | 11 |  |
| Hagovská & Olekszyová, 2016 | X | X | X | X | X | - | X | X | - | X | X | 8 |
| Conradsson et al., 2015 | X | X | X | X | - | - | - | X | - | X | X | 6 |
| Conradsson et al., 2019 | X | X | - | X | - | - | - | - | - | X | X | 4 |
| Silsupadol, Shumway-Cook, et al., 2009 | X | X | - | X | - | - | X | X | - | X | X | 6 |
| Lemke et al., 2019 | X | X | - | X | - | - | X | X | X | X | X | 7 |
| Wallen et al., 2018 | X | X | - | X | - | - | - | X | X | X | X | 6 |
| Makizako et al., 2012 | X | X | - | X | - | - | X | X | - | X | X | 6 |
| Silsupadol, Lugade, et al., 2009 | X | X | - | - | - | - | X | X | X | X | X | 6 |
| You et al., 2009 | X | X | - | X | - | - | - | - | - | - | X | 3 |
| Azadian et al., 2016 | - | X | - | X | - | - | - | X | - | X | X | 5 |
| Her et al., 2011 | X | X | - | X | - | - | - | X | - | X | X | 5 |
| Combourieu Donnezan et al., 2018 | X | X | - | X | - | - | - | X | - | X | X | 5 |
| Lipardo et al, 2020 | X | X | X | X | - | - | X | - | X | X | X | 7 |
| Uzunkulaoglu et al, 2020 | - | X | - | X | - | - | - | - | - | X | X | 4 |
| Schwenk et al., 2010 | X | X | X | X | X | - | - | - | - | X | X | 6 |
| Aydoğdu et al., 2018 | X | X | - | X | - | - | - | - | - | X | X | 4 |
| Bruno et al., 2017 | X | X | - | X | - | - | - | - | - | X | X | 4 |
| Ferraz et al., 2018 | X | X | X | X | - | - | X | X | - | X | X | 7 |
| Taylor et al., 2018 | X | X | X | X | - | - | X | X | - | X | X | 7 |
| Carpinella et al., 2017 | X | X | - | X | - | - | X | X | - | X | X | 6 |
| Liao et al., 2019 | X | X | X | X | - | - | X | X | - | X | X | 7 |
| Delbroek et al., 2017 | X | X | - | X | - | - | X | - | - | - | X | 4 |
| Daniel, 2012 | X | X | - | X | - | - | - | X | - | - | X | 4 |
| Szturm et al., 2011 | X | X | X | X | - | - | X | X | - | X | X | 7 |
| Padala et al., 2017 | X | X | X | X | - | - | - | - | X | X | X | 6 |
| Swinnen et al., 2021 | X | X | - | X | - | - | X | - | - | X | X | 5 |
| Pompeu et al., 2012 | - | X | - | X | - | - | X | - | - | X | X | 5 |
| Lee & Shin, 2013 | X | X | - | X | - | - | - | X | - | X | X | 5 |
| Mirelman et al., 2016 | X | X | X | X | - | - | X | X | X | X | X | 8 |
| Moreira et al., 2020 | X | X | - | X | - | - | - | - | - | X | X | 4 |

*1- eligibility criteria were specified, 2-subjects were randomly allocated to groups, 3-allocation was concealed, 4-the groups were similar at baseline regarding the most important prognostic indicators, 5-there was blinding of all subjects, 6-there was blinding of all therapists who administered the therapy, 7-there was blinding of all assessors who measured at least one key outcome, 8-measures of at least one key outcome were obtained from more than 85% of the subjects initially allocated to groups, 9-all subjects from whom outcome measures were available received the treatment or control condition as allocated or, where this was not the case, data for at least one key outcome was analyzed by »intention to treat«, 10-the results of between-group statistical comparisons are reported for at least one key outcome, the study provides both point measures and measures of variability for at least one key outcome
