# Supplementary material for: Impact of Motor-Cognitive Interventions on Selected Gait and Balance Outcomes in Older Adults: A Systematic Review and Meta-Analysis of Randomized Controlled Trials
Source: Front Psychol. 2022 Jun 16;13:837710. doi: 10.3389/fpsyg.2022.837710 (PMC9245546; doi:10.3389/fpsyg.2022.837710)
Supplement: Supplementary file 2 [file Table_2.docx]

**Table 2**

Diseased older adults – sequential cognitive-motor training

| **Study** | **Disease description** | **Sample description** | **Experimental design and duration of trial period** | **Control design and duration of trial period** | **Outcomes and results** |
| --- | --- | --- | --- | --- | --- |
| Hagovská & Olekszyová, 2016 | Mild cognitive impairment | N = 80  (CON = 40, EXP = 40)  Mean age_CON_ = 65.7 ± 5.6  Mean age_EXP_ = 68.2 ± 6.7 | In addition to the balance training participants performed cognitive task in Cogniplus program  PA training: 10 weeks: 7-times per week (30 min/trial)  Cognitive training: 10 weeks: 2-times per week (20 training sessions) | Balance training  10 weeks: 7-times per week (30 min/trial) | TUG |
