# Supplementary material for: Impact of Motor-Cognitive Interventions on Selected Gait and Balance Outcomes in Older Adults: A Systematic Review and Meta-Analysis of Randomized Controlled Trials
Source: Front Psychol. 2022 Jun 16;13:837710. doi: 10.3389/fpsyg.2022.837710 (PMC9245546; doi:10.3389/fpsyg.2022.837710)
Supplement: Supplementary file 3 [file Table_3.docx]

**Table 3**

Diseased older adults – simultaneous cognitive-motor training with additional cognitive task

| **Study** | **Disease description** | **Sample description** | **Experimental design and duration of trial period** | **Control design and duration of trial period** | **Outcomes and results** |
| --- | --- | --- | --- | --- | --- |
| Conradsson et al., 2015 | Parkinson disease | N = 91  (CON =44, EXP = 47)  Mean age_CON_ = 73.6 ± 5.3  Mean age_EXP_ = 72.9 ± 6.0 | Balance training while simultaneously performing cognitive tasks (HiBalance program)  10 weeks: 3-times per week (60 min/trial) | Passive group (only pre- and post-intervention assessment) | Gait speed – ↑ for EXP group  Gait speed -DT – ↑ for EXP group |
| Conradsson et al., 2019 | Osteoporosis | N = 68  (CON = 25, EXP = 43)  Mean age_CON_ = 76.0 ± 5.0  Mean age_EXP_ = 76.0 ± 6.0 | Balance exercise and motor tasks while simultaneously performing cognitive task.  12 week, 3-times per week (min 45 min/trial) | Passive group (only pre- and post-intervention assessment) | Gait speed – ↑ for EXP group |
| Silsupadol, Shumway-Cook, et al., 2009 | Balance impairment – less than 52 points on BBS-test | N = 15  (CON = 7, EXP = 8)  Mean age_CON_ = 74.7 ± 7.8  Mean age_EXP_ = 74.4 ± 6.2 | Balance training while simultaneously performing cognitive tasks.  4 weeks: 3-times per week (45 min/trial) | Balance training  4 weeks: 3-times per week (45 min/trial) | Gait speed – ↑ for CON and EXP group,  Gait speed - DT – ↑ for EXP group,  BBS – ↑ for CON group |
| Lemke et al., 2019 | Dementia | N = 105  (CON = 49, EXP = 56)  Mean age_CON_ = 82.6 ± 5.8  Mean age_EXP_ = 82.7 ± 6.2 | Walking training (+ exergame) while simultaneously performing cognitive tasks.  10 weeks: 2-times per week (90 min/trial) | Unspecific low-intensity training  10 weeks: 2-times per week (60 min/trial) | Gait speed - DT – ↑ for EXP group, |
| Wallen et al., 2018 | Parkinson disease | N = 100  (CON = 49, EXP = 51)  Mean age_CON_ = 73.0 ± 5.5  Mean age_EXP_ = 73.1 ± 5.8 | Balance and gait training while simultaneously performing cognitive tasks (HiBalance program)  10 weeks: 3-times per week (90 min/trial) | Passive group (only pre- and post-intervention assessment) | Gait speed – ↑ for EXP group |
| Makizako et al., 2012 | Mild cognitive impairment | N = 50  (CON = 25, EXP = 25)  Mean age_CON_ = 76.8 ± 6.8  Mean age_EXP_ = 75.3 ± 7.5 | Aerobic, strength and postural balance training while simultaneously performing cognitive tasks.  24 weeks: 2-times per week (90 min/trial) | Passive group (only pre- and post-intervention assessment) | (max) Gait speed (no improvement) |
| Silsupadol, Lugade, et al., 2009 | Balance impairment – less than 52 points on BBS-test | N = 15  (CON = 7, EXP = 8)  Mean age_CON_ = 74.7 ± 7.8  Mean age_EXP_ = 74.4 ± 6.2 | Balance training while simultaneously performing cognitive tasks.  4 weeks: 3-times per week (45 min/trial) | Balance training  4 weeks: 3-times per week (45 min/trial) | Gait speed (no improvement)  Gait speed - DT (no improvement) |
| You et al., 2009 | older adults with a history of falls | N = 13  (CON = 5, EXP = 8)  Mean age_CON_ = 68.0 ± 3.3  Mean age_EXP_ = 70.5 ± 6.8 | Walking training while simultaneously performing cognitive tasks.  6 weeks: 5-times per week (30 min/trial) | Walking training (with simple music - placebo)  6 weeks: 5-times per week (30 min/trial) | Gait speed – ↑ CON group |
| Azadian et al., 2016 | Balance impairment – less than 52 points on BBS-test | N = 30  (CON motor = 10, CON passive = 10, EXP = 10)  Mean age_CON motor_ = 73.8 ± 3.9 Mean age_CON_ _passive_ = 73.7 ± 4.4  Mean age_EXP_ = 73.9 ± 5.5 | Simple motor tasks while simultaneously performing cognitive tasks.  8 weeks: 3-times per week (45 min/trial) | CON motor = Executive functioning training  CON passive = Passive group (only pre- and post-intervention assessment)  8 weeks: 3-times per week (45 min/trial) | Gait speed – ↑ CON motor group |
| Her et al., 2011 | Stroke patients | N = 25  (CON motor = 12, EXP = 13)  Mean age_CON motor_ = 64.8 ± 5.2  Mean age_EXP_ = 64.5 ± 4.8 | Posture and balance training while simultaneously performing cognitive tasks.  6 weeks: 3-times per week (30 min/trial) | CON motor = Balance training while simultaneously performing different motor tasks.  6 weeks: 3-times per week (30 min/trial) | BBS (no improvement) |
| Combourieu Donnezan et al., 2018 | Mild cognitive impairment | N = 53  (CON motor = 18, CONpassive = 14, EXP = 21)  Mean age_CON motor_ = 77.1 ± 1.4  Mean age_CON passive_ = 79.2 ± 4  Mean age_EXP_ = 75.2 ± 1.3 | Bike training while simultaneously performing cognitive tasks.(aimed to stimulate attention and executive functions)  12 weeks: 2-times per week (60 min/trial) | CON motor = Aerobic bike training  CON passive = Passive group (only pre- and post-intervention assessment)  12 weeks: 2-times per week (60 min/trial) | Gait speed – ↑ for EXP group  Gait speed – DT ↑ for EXP group  TUG – ↑ for CON motor and EXP group |
| Lipardo et al, 2020 | Mild cognitive impairment | N = 69  (CON motor = 23, CONpassive = 23, EXP = 23)  Mean age_CON motor_ = 73 ± 7  Mean age_CON passive_ = 68 ± 8.5  Mean age_EXP_ = 67 ± 8 | Balance, strength, endurance, and flexibility training while simultaneously performing cognitive tasks (set of paper based cognitive exercises on executive function, memory, attention and orientation)  12 weeks: 3-times per week (60-90 min/trial) | CON motor = Balance, strength, endurance, and flexibility training  CON passive = Passive group (only pre- and post-intervention assessment)  12 weeks: 3-times per week (60-90 min/trial) | Gait speed (no improvement)  TUG – ↑ for EXP group |
| Uzunkulaoglu et al, 2020 | Osteoarthritic patients with balance impairment | N = 50  (CON = 25, EXP = 25)  Mean age_CON_ = 73.6 ± 5.6  Mean age_EXP_ = 72.3 ± 5.5 | Balance training while simultaneously performing cognitive tasks.  4 weeks: 3-times per week (min 45 min/trial) | Balance training  4 weeks: 3-times per week (min 45 min/trial) | Gait speed ↑ for CON and EXP group  Gait speed - DT↑ for CON and EXP group  TUG – ↑ for CON and EXP group  BBS – ↑ for CON and EXP group |
| Schwenk et al., 2010 | Dementia | N = 61  (CON = 35, EXP = 26)  Mean age_CON_ = 82.3 ± 7.9  Mean age_EXP_ = 80.4 ± 7.1 | Functional balance exercises while simultaneously performing cognitive tasks.  12 weeks: 2-times per week (min 120 min/trial) | Unspecific low-intensity training  12 weeks: 2-times per week (min 120 min/trial) | Gait speed  Gait speed -DT |
| Aydoğdu et al., 2018 | stroke patients | N = 53  (CON = 28, EXP = 25)  Mean age_CON_ = 71.2 ± 4.9  Mean age_EXP_ = 69.3 ± 5.0 | Walking training while simultaneously performing cognitive tasks.  8 weeks: 5-times per week (min 30 min/trial) | Walking training  8 weeks: 5-times per week (min 30 min/trial) | BBS –↑ for CON and EXP group |
| Bruno et al., 2017 | Hospitalized patients | N = 21  (CON = 11, EXP = 10)  Mean age_CON_ = 81 ± 6  Mean age_EXP_ = 81 ± 6 | Gait training combined with cognitive task (counting back  with different steps, recite the alphabet, etc.)  1 weeks: 5-times per week (30 min/trial) | Conventional physiotherapy  1 weeks: 5-times per week (30 min/trial) | Gait speed -DT (no improvement)  TUG (no improvement)  BBS (no improvement) |
