# Supplementary material for: Impact of Motor-Cognitive Interventions on Selected Gait and Balance Outcomes in Older Adults: A Systematic Review and Meta-Analysis of Randomized Controlled Trials
Source: Front Psychol. 2022 Jun 16;13:837710. doi: 10.3389/fpsyg.2022.837710 (PMC9245546; doi:10.3389/fpsyg.2022.837710)
Supplement: Supplementary file 4 [file Table_4.docx]

**Table 4**

Diseased older adults – exergaming

| **Study** | **Disease description** | **Sample description** | **Experimental design and duration of trial period** | **Control design and duration of trial period** | **Outcomes and results** |
| --- | --- | --- | --- | --- | --- |
| Ferraz et al., 2018 | Parkinson disease | N = 62  (CON motor = 22, CON bicycle = 20, EXP = 20)  Mean age_CON motor_ = 71.0 ± /  Mean age_CON bicycle_ = 67.0 ± /  Mean age_EXP_ = 67.0 ± / | Xbox 360 Kinect  8 weeks: 3-times per week (50 min/trial) | CON motor = functional training  CON bicycle = bicycle exercise  8 weeks: 3-times per week (50 min/trial) | Gait speed – ↑ for EXP group |
| Taylor et al., 2018 | Long-term care residents | N = 58  (CON = 32, EXP = 25)  Mean age_CON_ = 85.8 ± 7.2  Mean age_EXP_ = 86.7 ± 7.4 | Xbox 360 Kinect  8 weeks: 2-times per week (35 min/trial) | Passive group (only pre- and post-intervention assessment) | TUG (no improvement) |
| Carpinella et al., 2017 | Parkinson disease | N = 37  (CON = 20, EXP = 17)  Mean age_CON_ = 75.6 ± 8.2  Mean age_EXP_ = 73.0 ± 7.1 | Balance and gait tailored exercises included within Gamepad.  7 weeks: 3-times per week (45 min/trial) | Balance and gait exercises  7 weeks: 3-times per week (45 min/trial) | Gait speed (no improvement)  BBS – ↑ for EXP group  TUG (no improvement) |
| Bruno et al., 2017 | Hospitalized patients | N = 21  (CON = 11, EXP = 10)  Mean (age_CON;_ age_EXP_) = 81.0 ± 6.0 | Nintendo Wii Balance Board  1 weeks: 5-times per week (30 min/trial) | Conventional physiotherapy  1 weeks: 5-times per week (30 min/trial) | Gait speed -DT (no improvement)  TUG (no improvement)  BBS – ↑ for EXP and CON group |
| Liao et al., 2019 | Mild cognitive impairment | N = 52  (CON = 25, EXP = 27)  Mean age_CON_ = 84.1 ± 5.5  Mean age_EXP_ = 79.6 ± 8.5 | Kinect-based exergaming  12 weeks: 3-times per week (60 min/trial) | Combined exercise training  12 weeks: 3-times per week (60 min/trial) | Gait speed – ↑ for EXP and CON group  TUG – ↑ for EXP group |
| Delbroek et al., 2017 | Mild cognitive impairment | N = 17  (CON = 9, EXP = 8)  Mean age_CON_ = 87.5 ± 6.6  Mean age_EXP_ = 86.9 ± 5.6 | Virtual reality dual-task training using the BioRescue.  6 weeks: 2-times per week (25 min/trial) | Passive group (only pre- and post-intervention assessment) | TUG – ↑ for EXP group |
| Daniel, 2012 | Frailty as defined by Fried et al. (2001) | N = 23  (CON motor = 8, CON passive = 7, EXP = 8)  Mean age_CON motor_ = 78.1 ± 5.5  Mean age_CON passive_ = 72.6 ± 4.6  Mean age_EXP_ = 80.0 ± 3.4 | Nintendo Wii-Fit  15 weeks: 3-times per week (45 min/trial) | CON motor = Traditional senior fitness program, and a rigorous seated aerobics program.  CON passive = Passive group (only pre- and post-intervention assessment)  15 weeks: 3-times per week (45 min/trial) | Gait speed – ↑ for CON motor and EXP group  TUG |
| Szturm et al., 2011 | Balance impairment and mobility limitations | N = 27  (CON = 13, EXP = 14)  Mean age_CON_ = 81.0 ± 7.0  Mean age_EXP_ = 80.5 ± 6 | Balance exercises coupled with video game play.  8 weeks: 2-times per week (45 min/trial) | Rehabilitation program consisting of strengthening and balance exercises.  8 weeks: 2-times per week (45 min/trial) | BBS – ↑ for EXP and CON group  TUG (no improvement)  Gait speed (no improvement) |
| Padala et al., 2017 | Mild Alzheimer's disease | N = 30  (CON = 15, EXP = 15)  Mean age_CON_ = 73.9 ± 7.1  Mean age_EXP_ = 72.1 ± 5.3 | Nintendo Wii-Fit  8 weeks: 5-times per week (30 min/trial) | Walking training  8 weeks: 5-times per week (30 min/trial) | BBS – ↑ for EXP and CON group |
| Swinnen et al., 2021 | Major neurocognitive disorder | N = 45  (CON = 22, EXP = 23)  Mean age_CON_ = 85.3 ± 6.5  Mean age_EXP_ = 84.7 ± 5.6 | Exergame device “Dividat Senso”  8 weeks: 3-times per week (15 min/trial) | Passive group (only pre- and post-intervention assessment) | Gait speed – ↑ for EXP group |
| Pompeu et al., 2012 | Parkinson disease | N = 45  (CON = 22, EXP = 23)  Mean age_CON_ = 67.4 ± 8.1  Mean age_EXP_ = 67.4 ± 8.1 | Nintendo Wii-Fit  7 weeks: 2-times per week (30 min/trial) | Balance training  7 weeks: 2-times per week (30 min/trial) | BBS – ↑ for EXP and CON group |
| Lee & Shin, 2013 | Older adults with Diabetes Mellitus | N = 282  (CON = 28, EXP = 27)  Mean age_CON_ = 74.3 ± 5.2  Mean age_EXP_ = 73.8 ± 4.8 | PlayStation 2 (EyeToy)  10 weeks: 2-times per week (50 min/trial) | Passive group (only pre- and post-intervention assessment) | Gait speed – ↑ for EXP group  BBS – ↑ for EXP group  TUG – ↑ for EXP group |

| Mirelman et al., 2016 | Older adults with varied motor and cognitive deficits | N = 55  (CON = 136, EXP = 146)  Mean age_CON_ = 73.3 ± 6.4  Mean age_EXP_ = 74.2 ± 6.9 | Treadmill training VR  6 weeks: 3-times per week (45 min/trial) | Treadmill training  6 weeks: 3-times per week (45 min/trial) | Gait speed – ↑ for EXP group |
| --- | --- | --- | --- | --- | --- |
| Moreira et al., 2021 | Older adults classified as pre-frail | N = 66 (female)  (CON = 34, EXP = 32)  Mean age_CON_ = 70.8 ± 5.6  Mean age_EXP_ = 70.8 ± 4.5 | Xbox 360 Kinect  12 weeks: 3-times per week (50 min/trial) | Strength, balance, and cardiorespiratory training  12 weeks: 3-times per week (50 min/trial) | Gait speed – ↑ for CON group  TUG – ↑ for EXP and CON group |
