# Supplementary figures and images for: Impact of Motor-Cognitive Interventions on Selected Gait and Balance Outcomes in Older Adults: A Systematic Review and Meta-Analysis of Randomized Controlled Trials
Source: Front Psychol. 2022 Jun 16;13:837710. doi: 10.3389/fpsyg.2022.837710 (PMC9245546; doi:10.3389/fpsyg.2022.837710)

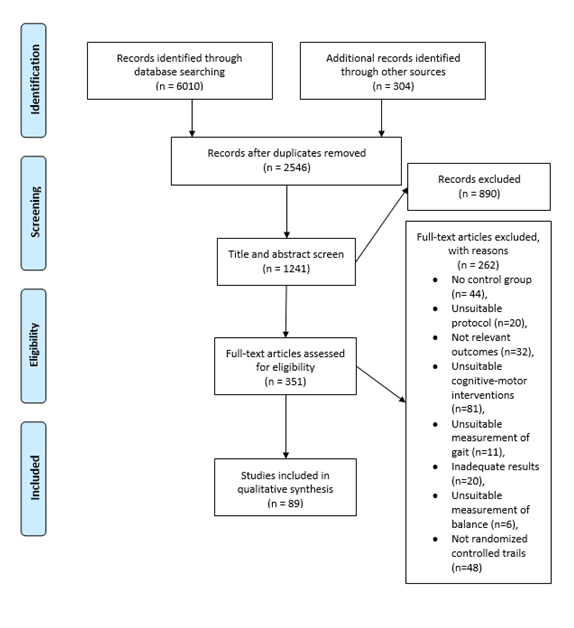

Supplement: Supplementary file 5 [file Image_1.png]

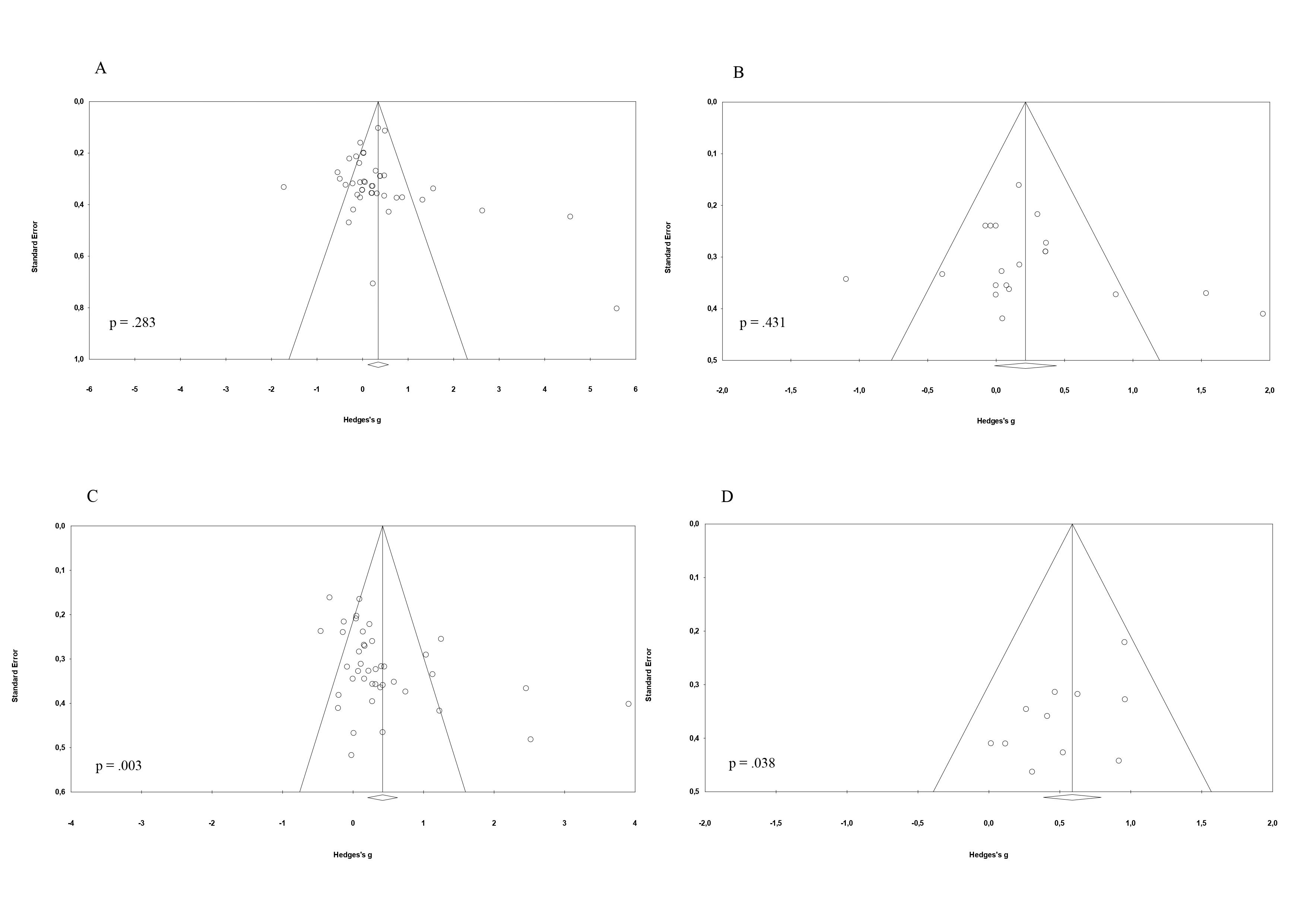

Supplement: Supplementary file 6 [file Image_2.jpg]
