# Supplementary material for: Impact of Motor-Cognitive Interventions on Selected Gait and Balance Outcomes in Older Adults: A Systematic Review and Meta-Analysis of Randomized Controlled Trials
Source: Front Psychol. 2022 Jun 16;13:837710. doi: 10.3389/fpsyg.2022.837710 (PMC9245546; doi:10.3389/fpsyg.2022.837710)
Supplement: Supplementary file 7 [file Data_Sheet_1.docx]

**REFERENCE LIST FOR ALL CITED REFERENCES IN THE SUPPLEMENTARY LIST**

Azadian, E. *et al.* (2016) ‘The effect of dual task and executive training on pattern of gait in older adults with balance impairment: A Randomized controlled trial’, *Archives of Gerontology and Geriatrics*, 62, pp. 83–89. doi:10.1016/j.archger.2015.10.001.

Bruno, B. *et al.* (2017) ‘A preliminary study of the integration of specially developed serious games in the treatment of hospitalized elderly patients’, in *2017 International Conference on Virtual Rehabilitation (ICVR)*. *2017 International Conference on Virtual Rehabilitation (ICVR)*, Montreal, QC, Canada: IEEE, pp. 1–6. doi:10.1109/ICVR.2017.8007508.

Carpinella, I. *et al.* (2017) ‘Wearable Sensor-Based Biofeedback Training for Balance and Gait in Parkinson Disease: A Pilot Randomized Controlled Trial’, *Archives of Physical Medicine and Rehabilitation*, 98(4), pp. 622-630.e3. doi:10.1016/j.apmr.2016.11.003.

Conradsson, D. *et al.* (2015) ‘The Effects of Highly Challenging Balance Training in Elderly With Parkinson’s Disease: A Randomized Controlled Trial’, *Neurorehabilitation and Neural Repair*, 29(9), pp. 827–836. doi:10.1177/1545968314567150.

Conradsson, D. and Halvarsson, A. (2019) ‘The effects of dual-task balance training on gait in older women with osteoporosis: A randomized controlled trial’, *Gait & Posture*, 68, pp. 562–568. doi:10.1016/j.gaitpost.2019.01.005.

Daniel, K. (2012) ‘Wii-Hab for Pre-Frail Older Adults’, *Rehabilitation Nursing*, 37(4), pp. 195–201. doi:10.1002/rnj.25.

Delbroek, T., Vermeylen, W. and Spildooren, J. (2017) ‘The effect of cognitive-motor dual task training with the biorescue force platform on cognition, balance and dual task performance in institutionalized older adults: a randomized controlled trial’, *Journal of Physical Therapy Science*, 29(7), pp. 1137–1143. doi:10.1589/jpts.29.1137.

Ferraz, D.D. *et al.* (2018) ‘The Effects of Functional Training, Bicycle Exercise, and Exergaming on Walking Capacity of Elderly Patients With Parkinson Disease: A Pilot Randomized Controlled Single-blinded Trial’, *Archives of Physical Medicine and Rehabilitation*, 99(5), pp. 826–833. doi:10.1016/j.apmr.2017.12.014.

Hagovská, M. and Olekszyová, Z. (2016) ‘Impact of the combination of cognitive and balance training on gait, fear and risk of falling and quality of life in seniors with mild cognitive impairment: Fear and risk of falling in seniors’, *Geriatrics & Gerontology International*, 16(9), pp. 1043–1050. doi:10.1111/ggi.12593.

Her, J.-G. *et al.* (2011) ‘Effects of Balance Training with Various Dual-Task Conditions on Stroke Patients’, *Journal of Physical Therapy Science*, 23(5), pp. 713–717. doi:10.1589/jpts.23.713.

Lee, S. and Shin, S. (2013) ‘Effectiveness of Virtual Reality Using Video Gaming Technology in Elderly Adults with Diabetes Mellitus’, *Diabetes Technology & Therapeutics*, 15(6), pp. 489–496. doi:10.1089/dia.2013.0050.

Lemke, N.C. *et al.* (2019) ‘Transferability and Sustainability of Motor-Cognitive Dual-Task Training in Patients with Dementia: A Randomized Controlled Trial’, *Gerontology*, 65(1), pp. 68–83. doi:10.1159/000490852.

Liao, Y.-Y. *et al.* (2019) ‘Effects of Virtual Reality-Based Physical and Cognitive Training on Executive Function and Dual-Task Gait Performance in Older Adults With Mild Cognitive Impairment: A Randomized Control Trial’, *Frontiers in Aging Neuroscience*, 11, p. 162. doi:10.3389/fnagi.2019.00162.

Lipardo, D.S. and Tsang, W.W. (2020) ‘Effects of combined physical and cognitive training on fall prevention and risk reduction in older persons with mild cognitive impairment: a randomized controlled study’, *Clinical Rehabilitation*, 34(6), pp. 773–782. doi:10.1177/0269215520918352.

Makizako, H. *et al.* (2012) ‘Does a multicomponent exercise program improve dual-task performance in amnestic mild cognitive impairment? A randomized controlled trial’, *Aging Clinical and Experimental Research*, 24(6), pp. 640–646. doi:10.3275/8760.

Mirelman, A. *et al.* (2016) ‘Addition of a non-immersive virtual reality component to treadmill training to reduce fall risk in older adults (V-TIME): a randomised controlled trial’, *The Lancet*, 388(10050), pp. 1170–1182. doi:10.1016/S0140-6736(16)31325-3.

Moreira, N.B. *et al.* (2021) ‘Perceptive–Cognitive and Physical Function in Prefrail Older Adults: Exergaming Versus Traditional Multicomponent Training’, *Rejuvenation Research*, 24(1), pp. 28–36. doi:10.1089/rej.2020.2302.

Padala, K.P. *et al.* (2017) ‘Home-Based Exercise Program Improves Balance and Fear of Falling in Community-Dwelling Older Adults with Mild Alzheimer’s Disease: A Pilot Study’, *Journal of Alzheimer’s Disease*. Edited by M. Montero-Odasso, 59(2), pp. 565–574. doi:10.3233/JAD-170120.

Pompeu, J.E. *et al.* (2012) ‘Effect of Nintendo Wii^TM^-based motor and cognitive training on activities of daily living in patients with Parkinson’s disease: A randomised clinical trial’, *Physiotherapy*, 98(3), pp. 196–204. doi:10.1016/j.physio.2012.06.004.

Schwenk, M. *et al.* (2010) ‘Dual-task performances can be improved in patients with dementia: A randomized controlled trial’, *Neurology*, 74(24), pp. 1961–1968. doi:10.1212/WNL.0b013e3181e39696.

Silsupadol, P., Shumway-Cook, A., *et al.* (2009) ‘Effects of Single-Task Versus Dual-Task Training on Balance Performance in Older Adults: A Double-Blind, Randomized Controlled Trial’, *Archives of Physical Medicine and Rehabilitation*, 90(3), pp. 381–387. doi:10.1016/j.apmr.2008.09.559.

Silsupadol, P., Lugade, V., *et al.* (2009) ‘Training-related changes in dual-task walking performance of elderly persons with balance impairment: A double-blind, randomized controlled trial’, *Gait & Posture*, 29(4), pp. 634–639. doi:10.1016/j.gaitpost.2009.01.006.

Swinnen, N. *et al.* (2021) ‘The efficacy of exergaming in people with major neurocognitive disorder residing in long-term care facilities: a pilot randomized controlled trial’, *Alzheimer’s Research & Therapy*, 13(1), p. 70. doi:10.1186/s13195-021-00806-7.

Szturm, T. *et al.* (2011) ‘Effects of an Interactive Computer Game Exercise Regimen on Balance Impairment in Frail Community-Dwelling Older Adults: A Randomized Controlled Trial’, *Physical Therapy*, 91(10), pp. 1449–1462. doi:10.2522/ptj.20090205.

Taylor, L. *et al.* (2018) ‘Exergames to Improve the Mobility of Long-Term Care Residents: A Cluster Randomized Controlled Trial’, *Games for Health Journal*, 7(1), pp. 37–42. doi:10.1089/g4h.2017.0084.

Tetik Aydoğdu, Y., Aydoğdu, O. and İnal, H.S. (2018) ‘The Effects of Dual-Task Training on Patient Outcomes of Institutionalized Elderly Having Chronic Stroke’, *Dementia and Geriatric Cognitive Disorders Extra*, 8(3), pp. 328–332. doi:10.1159/000492964.

Uzunkulaoglu, A. *et al.* (2020) ‘Effects of singl-task versus dual-task training on balance performance in elderly patients with knee osteoarthritis’, *Arch Rheumatol*, 35(1), pp. 35–40. doi:10.5606/ArchRheumatol.2020.7174.

Wallén, M.B. *et al.* (2018) ‘Long-term effects of highly challenging balance training in Parkinson’s disease—a randomized controlled trial’, *Clinical Rehabilitation*, p. 026921551878433. doi:10.1177/0269215518784338.

You, J.H. *et al.* (2009) ‘Effects of dual-task cognitive-gait intervention on memory and gait dynamics in older adults with a history of falls: A preliminary investigation’, *NeuroRehabilitation*, 24(2), pp. 193–198. doi:10.3233/NRE-2009-0468.
